# Real-world federated learning in radiology: hurdles to overcome and benefits to gain

**DOI:** 10.1093/jamia/ocae259

**Published:** 2024-10-25

**Authors:** Markus Ralf Bujotzek, Ünal Akünal, Stefan Denner, Peter Neher, Maximilian Zenk, Eric Frodl, Astha Jaiswal, Moon Kim, Nicolai R Krekiehn, Manuel Nickel, Richard Ruppel, Marcus Both, Felix Döllinger, Marcel Opitz, Thorsten Persigehl, Jens Kleesiek, Tobias Penzkofer, Klaus Maier-Hein, Andreas Bucher, Rickmer Braren

**Affiliations:** Division of Medical Image Computing, German Cancer Research Center Heidelberg, Heidelberg, 69120, Germany; Medical Faculty Heidelberg, University of Heidelberg, Heidelberg, 69120, Germany; Division of Medical Image Computing, German Cancer Research Center Heidelberg, Heidelberg, 69120, Germany; Division of Medical Image Computing, German Cancer Research Center Heidelberg, Heidelberg, 69120, Germany; Faculty of Mathematics and Computer Science, Heidelberg University, Heidelberg, 69120, Germany; Division of Medical Image Computing, German Cancer Research Center Heidelberg, Heidelberg, 69120, Germany; Pattern Analysis and Learning Group, Department of Radiation Oncology, Heidelberg University Hospital, Heidelberg, 69120, Germany; German Cancer Consortium (DKTK), Partner Site Heidelberg, Heidelberg, 69120, Germany; Division of Medical Image Computing, German Cancer Research Center Heidelberg, Heidelberg, 69120, Germany; Medical Faculty Heidelberg, University of Heidelberg, Heidelberg, 69120, Germany; Institute for Diagnostic and Interventional Radiology, University Hospital Frankfurt, Frankfurt (Main), 60590, Germany; Goethe University Frankfurt, Frankfurt, 60590, Germany; Institute for Diagnostic and Interventional Radiology, Faculty of Medicine, University Hospital Cologne, University of Cologne, Cologne, 50937, Germany; Institute for AI in Medicine (IKIM), University Hospital Essen (AöR), Essen, 45131, Germany; Intelligent Imaging Lab@Section Biomedical Imaging, Department of Radiology and Neuroradiology, University Medical Center Schleswig-Holstein (UKSH), Kel, 24118, Germany; Institute for AI in Medicine, Technical University of Munich, Munich, 81675, Germany; Department of Radiology, Charité—Universitätsmedizin Berlin, Berlin, 10117, Germany; Department of Radiology and Neuroradiology, University Medical Centers Schleswig-Holstein, Kiel, 24105, Germany; Department of Radiology, Charité—Universitätsmedizin Berlin, Berlin, 10117, Germany; Institute for Diagnostic and Interventional Radiology and Neuroradiology, University Hospital Essen (AÖR), Essen, 45131, Germany; Institute for Diagnostic and Interventional Radiology, Faculty of Medicine, University Hospital Cologne, University of Cologne, Cologne, 50937, Germany; Institute for AI in Medicine (IKIM), University Hospital Essen (AöR), Essen, 45131, Germany; Department of Radiology, Charité—Universitätsmedizin Berlin, Berlin, 10117, Germany; Berlin Institute of Health, Berlin, 10178, Germany; Division of Medical Image Computing, German Cancer Research Center Heidelberg, Heidelberg, 69120, Germany; Pattern Analysis and Learning Group, Department of Radiation Oncology, Heidelberg University Hospital, Heidelberg, 69120, Germany; German Cancer Consortium (DKTK), Partner Site Heidelberg, Heidelberg, 69120, Germany; National Center for Tumor Diseases (NCT), NCT Heidelberg, A Partnership Between DKFZ and The University Medical Center Heidelberg, Heidelberg, 69120, Germany; Institute for Diagnostic and Interventional Radiology, University Hospital Frankfurt, Frankfurt (Main), 60590, Germany; Goethe University Frankfurt, Frankfurt, 60590, Germany; Institute for Diagnostic and Interventional Radiology, Klinikum rechts der Isar, Technical University of Munich, Munich, 81675, Germany

**Keywords:** radiology, artificial intelligence, federated learning, healthcare infrastructure, distributed systems

## Abstract

**Objective:**

Federated Learning (FL) enables collaborative model training while keeping data locally. Currently, most FL studies in radiology are conducted in simulated environments due to numerous hurdles impeding its translation into practice. The few existing real-world FL initiatives rarely communicate specific measures taken to overcome these hurdles. To bridge this significant knowledge gap, we propose a comprehensive guide for real-world FL in radiology. Minding efforts to implement real-world FL, there is a lack of comprehensive assessments comparing FL to less complex alternatives in challenging real-world settings, which we address through extensive benchmarking.

**Materials and Methods:**

We developed our own FL infrastructure within the German Radiological Cooperative Network (RACOON) and demonstrated its functionality by training FL models on lung pathology segmentation tasks across six university hospitals. Insights gained while establishing our FL initiative and running the extensive benchmark experiments were compiled and categorized into the guide.

**Results:**

The proposed guide outlines essential steps, identified hurdles, and implemented solutions for establishing successful FL initiatives conducting real-world experiments. Our experimental results prove the practical relevance of our guide and show that FL outperforms less complex alternatives in all evaluation scenarios.

**Discussion and Conclusion:**

Our findings justify the efforts required to translate FL into real-world applications by demonstrating advantageous performance over alternative approaches. Additionally, they emphasize the importance of strategic organization, robust management of distributed data and infrastructure in real-world settings. With the proposed guide, we are aiming to aid future FL researchers in circumventing pitfalls and accelerating translation of FL into radiological applications.

## Objectives

Deep learning (DL) has revolutionized radiological image analysis, rapidly driving radiological research and increasingly transforming clinical routine. Training powerful DL models requires access to vast and diverse datasets. A practical approach is to train on centralized data from multiple centers in a pooled data lake. However, such data aggregation is often complicated by various regulatory requirements, including privacy regulations such as GDPR, HIPAA, state-specific healthcare laws or federal privacy laws.^1^ Federated Learning (FL)^2^ resolves these issues by keeping data at the originating medical centers. Instead of sharing data, FL collaboratively trains models through periodic exchanges of DL model weights between locally training participants and a central server achieving performances comparable to centralized trained models.^3^ Consequently, FL holds significant potential in healthcare enabling sufficiently large datasets, even for rare diseases or minority populations.^4^

Most FL research is currently conducted in simulated environments,^5^ lacking broad translation into real-world applications due to practical implementation challenges. To address this predicament, we identified three major problems (*P*). *P1*: The few studies examining real-world FL in general medicine,^6–11^ and specifically in radiology,^4^^,^^7^^,^^12–18^ provide limited insights into real-world challenges of implementing FL in practice, leaving a significant knowledge gap. *P2*: Theoretical FL studies,^5^^,^^12^^,^^19–34^ which discuss hypothetical applications and potential challenges of FL in medicine, often do not account for real-world complexities and lack real-world FL investigations proving actual experience with these challenges, thereby reducing their practical relevance. *P3*: Given the numerous real-world challenges of FL, one may question its benefits or lean towards less complex alternatives. However, there is a lack of comprehensive benchmarking comparing FL to alternatives in a real-world setting across various evaluation scenarios.

Within the framework of the German Radiological Cooperative Network (RACOON) (https://racoon.network/),^35^ we have built and established the first nation-wide collaborative radiology initiative of its kind, that includes all 38 university hospitals of the country. We evaluated its functionality by conducting proof-of-concept real-world FL experiments to collaboratively train DL segmentation models on radiological image data across six university hospitals. This setup provides a ready-to-use infrastructure for future researchers to conduct clinical research using FL without building their own systems. During developing this FL initiative and conducting experiments with real-world datasets in a real-world setting, we encountered and overcame numerous difficulties and practical hurdles. These novel insights, largely unknown in the literature,^4–9^^,^^12–16^^,^^19–34^ motivated us to compile an extensive guide for real-world FL in radiology.

**Figure 1.**
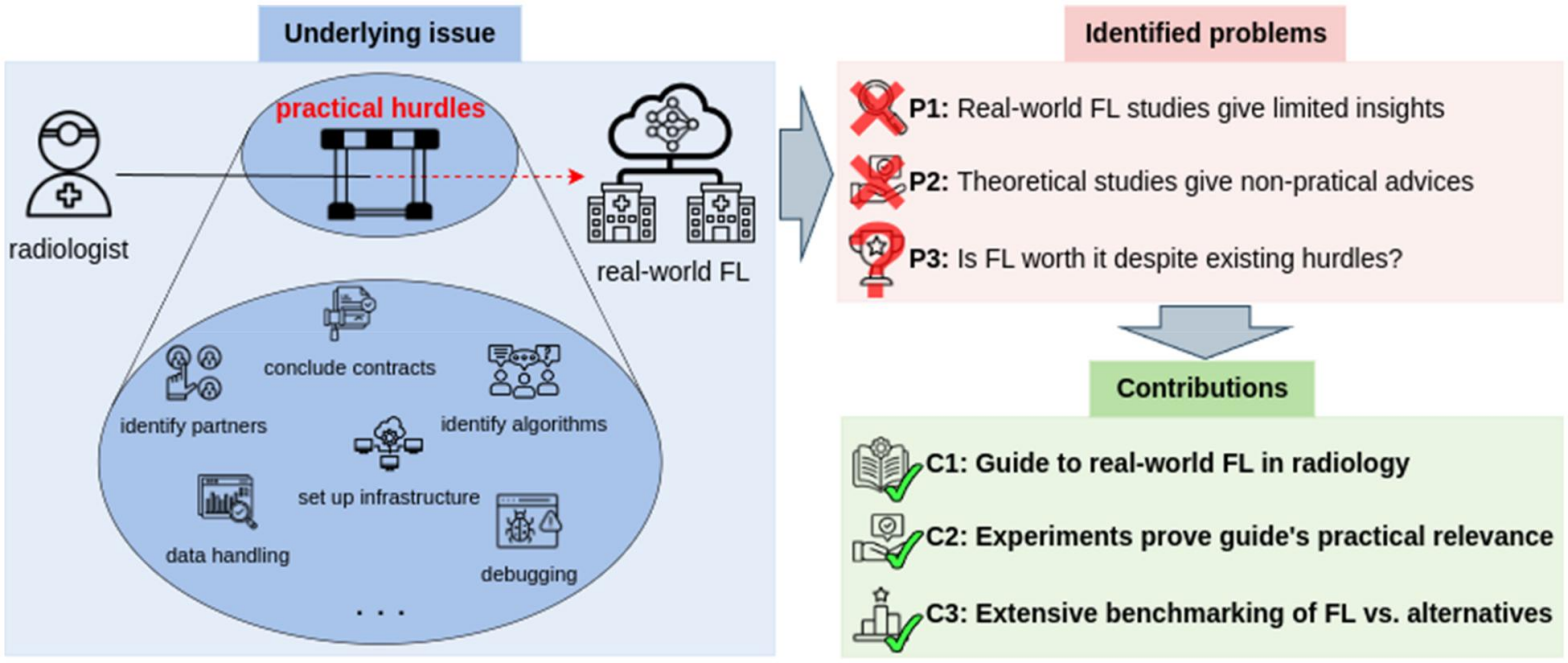
The underlying issue, three identified problems and corresponding, solving contributions to establish real-world FL initiatives in radiology. The underlying issue is that practical hurdles and inherent difficulties impede a straightforward realization of FL in the real world. We identified with *P1*-*P3* three major problems and aim to solve them with our contributions *C1*-*C3*.

Our contributions (*C*) tackling the identified problems (*P*) are threefold, see Figure 1. *C1*: We propose a detailed guide for building real-world FL initiatives based on our first-hand experiences along with relevant literature. This guide outlines essential steps, highlights encountered issues and provides solutions for each phase of real-world FL in radiological research, aiming to support and accelerate future efforts. *C2*: We conducted real-world training of DL segmentation models for lung pathology detection using data distributed across six sites, demonstrating the functionality of our FL infrastructure, and emphasizing the practical relevance of our proposed guide. *C3*: Recognizing the challenges of real-world FL (*C1*) and the potential preference for less complex approaches, we compare FL to simpler alternatives like local model training and ensembling.^36–38^ We benchmark these approaches extensively across various evaluation scenarios: personalization, ie benefits participating sites gain from FL training; and generalization, ie benefits non-participating sites gain from leveraging collaboratively trained models from other sites, with or without incorporating local training capabilities.

## Materials and methods

### Categorization of insights in building real-world FL initiatives

The term “challenge” is frequently used in FL research, yet the issues encountered in actual real-world FL implementations are diverse. We define “real-world” studies as those utilizing private, sensitive datasets that are not publicly available and employing distributed computing infrastructure integrated into clinical IT ecosystems. Additionally, we characterize a FL initiative as the combination of community engagement, organizational structures, legal agreements, and infrastructure necessary to conduct and scale FL experiments beyond single execution.

For our proposed guide (*C1*), we classify challenges of real-world FL implementation into two categories: practical hurdles and inherent difficulties. *Practical Hurdles* encompass organizational, legal, or technical issues that are solved through agreements or technical solutions. We share our solutions based on practical experiences with these hurdles. In contrast, inherent *difficulties* refer to limitations in real-world FL of organizational, technical, or research-related nature that cannot be avoided but must be acknowledged when successfully developing FL initiatives and conducting real-world experiments.

We further categorize these issues based on the scope within which they impact the establishment of a FL initiative. These categories include organization, legal requirements, infrastructure setup, experiment preparation, and experiments and evaluation.

### Real-world FL study: experimental setup

#### FL infrastructure

Our real-world FL efforts are part of the German RACOON initiative, which aims to use artificial intelligence to advance radiological research across all 38 German university hospitals. Each hospital is equipped with a server hosting key software: Mint Lesion (https://mint-medical.com/de/mint-lesion) for structured radiological reporting, SATORI (https://www.mevis.fraunhofer.de/de/research-and-technologies/werkzeuge-fuer-ki-kollaborationen.html) and ImFusion Labels (https://www.imfusion.com/products/imfusion-labels) for imaging data annotation, and a Kaapana-based (https://www.kaapana.ai/) platform for medical image processing. Thereby, Kaapana facilitates curating radiological data^39^ for subsequent local and federated training^38^ of DL models, supporting various studies within RACOON.

**Figure 2.**
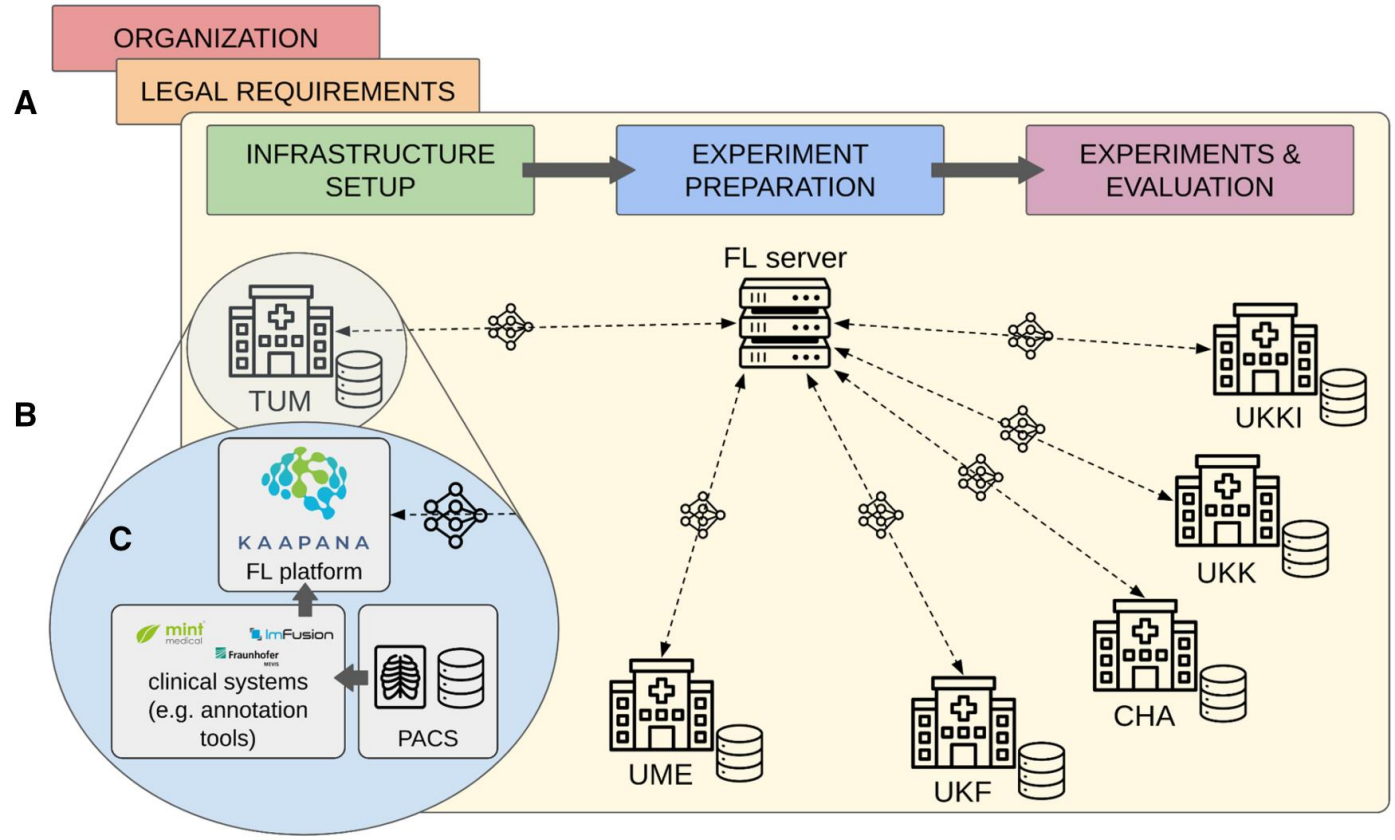
Phases of the proposed guide for building and deploying real-world FL in radiology (A) and the infrastructure of the RACOON FL initiative (B). FL infrastructure with a central FL server and six participating sites (TUM, UME, UKF, CHA, UKK, UKKI) maintaining data locally and periodically exchanging model weights during FL training. Detailed view of the site infrastructure (C): radiological images are queried from the PACS, processed by third party clinical systems (e.g, annotation tools) and used for FL training using the FL platform.

In our real-world FL experiments, we orchestrated a centralized FL initiative across a subset of six out of 38 university hospitals by connecting their Kaapana platforms to a central server. The six university hospitals were: Charité Berlin (CHA), Technical University of Munich (TUM), University Medicine Essen (UME), and the university hospitals in Frankfurt am Main (UKF), Cologne (UKK) and Kiel (UKKI) (Figure 2).

#### Dataset

**Figure 3.**
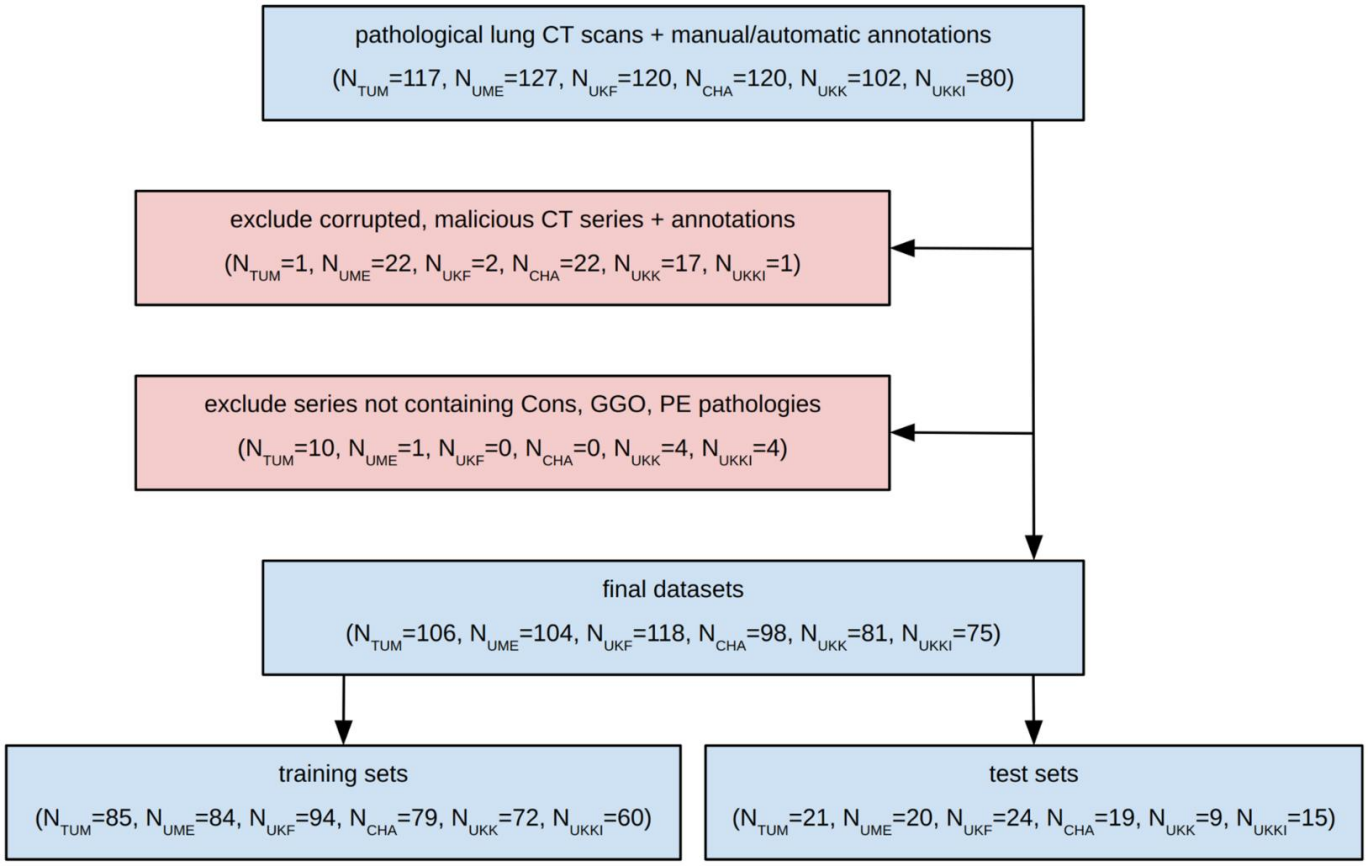
Cohort definition after data curation, filtering and splitting of the distributed data across the six participating sites resulting in the final training and test sets.

**Figure 4.**
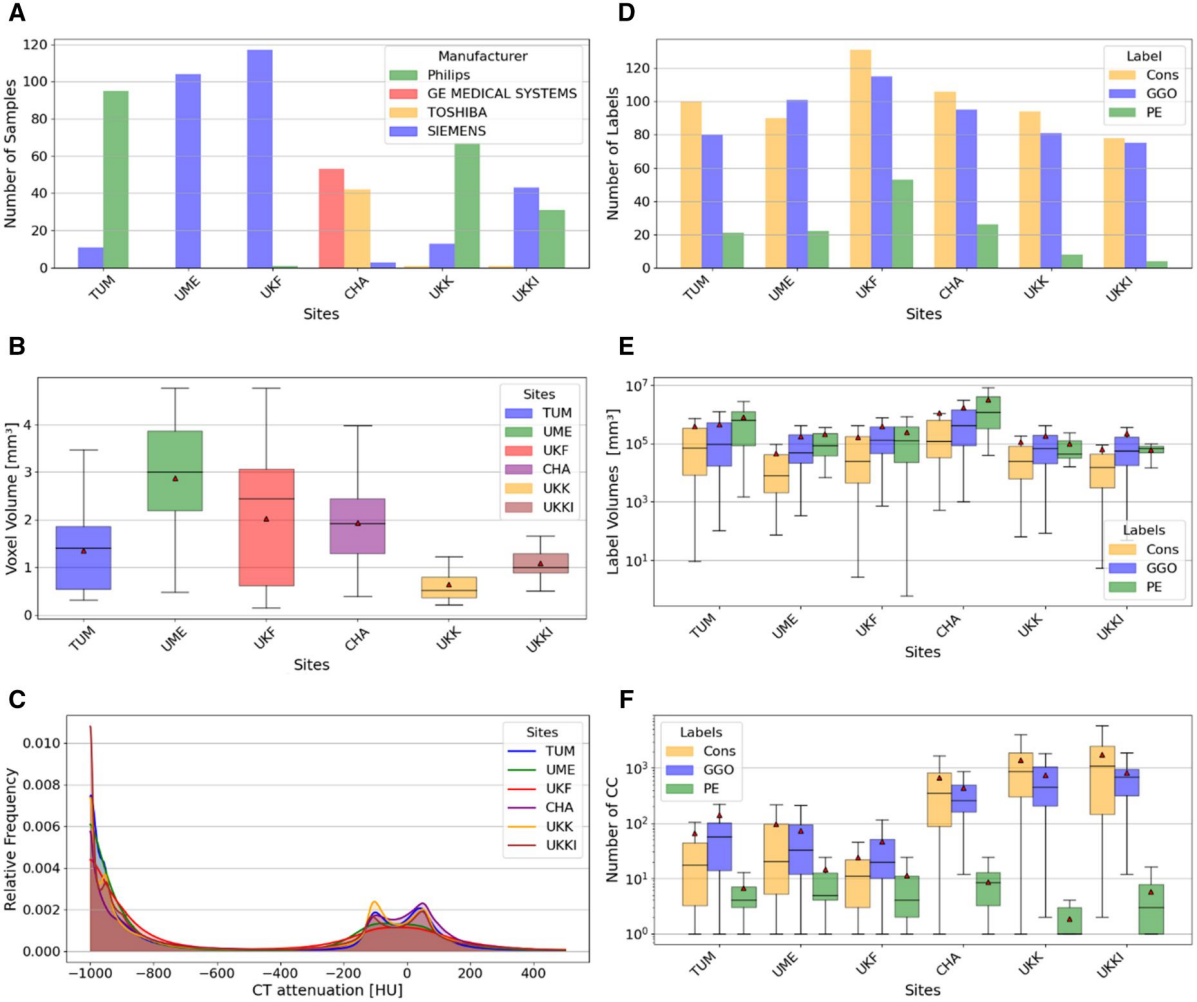
Data characteristics of CT data and annotations labels across the six participating sites. (A) CT scanner manufacturer distribution; (B) Average voxel volume distribution of CT scans; (C) Relative histogram of CT attenuation in HU (Note: -1000 HU visualizing air); (D) Annotation label distribution; (E) Annotation label volume distribution; (F) CCA: Number of CC per annotation label.

In our FL experiments (*C2*), we trained a DL segmentation model on a lung CT dataset where every scan contains pathologies, due to the cohort design investigating various lung diseases based on the extent of pathologies. For this dataset voxel-level segmentation annotations of three pathologies were created by independent radiological readers, supervised by experienced board-certified radiologists at each site. The three types of pathological image patterns segmented were: (non-)malignant consolidation (Cons), ground-glass opacity (GGO), and pleural effusion (PE), which are significant predictors of disease progression in various lung diseases.^40^ To avoid bias towards specific diseases, the dataset was curated to maintain a balanced number of samples from 20 different lung diseases across all sites. Data provision varied between sites: TUM, UME, and UKF provided manually generated voxel-level annotations; sites CHA, UKK, and UKKI provided automatically pre-processed, manually corrected annotations created using a nnU-Net model^41^ trained on public data^42^ (qualitative annotation comparison in Figure A1). For model training, data at each site was split into training and test sets with an 80% to 20% ratio, with special care taken to maintain this ratio for less common PE cases. Following this data-splitting and site-specific inclusion and exclusion criteria, resulted in curated datasets per site as shown in Figure 3. Detailed descriptive statistics of the distributed data given in Figure 4.

#### Training details

The DL model utilized is a state-of-the-art U-Net model from the self-configuring medical image segmentation framework nnU-Net.^41^^,^^43^ The self-configuration process of the nnU-Net model uses a dataset fingerprint optimizing the model configuration through rule-based, fixed, and empirical parameter selection, making it a well-performing off-the-shelf baseline model.

The model's self-configuration process is straightforward for local training. However, in a federated setup, this procedure requires multiple steps to synchronize across participating sites, following the implementation in Kades et al.^38^ Each site generates a dataset fingerprint from its local training data, which is sent to the central server. The server aggregates these fingerprints and redistributes them to all sites, ensuring each site configures and initializes the model identically (Figure A2).

In our experiments, we used the low-resolution configuration of the nnU-Net model to optimize training efficiency and retained its self-configured parameters without further modifications or hyper-parameter tuning. Consequently, there was no need for a validation split and we trained the model for a fixed number of 1000 epochs (ideal for local training^41^) Each model processed 250 batches per epoch; further nnU-Net training details provided in Table A1. To optimize all site’s objectives equally, we utilized non-weighted averaging updating global model weights $w_{\mathrm{glob}}$ from locally updated model weights $w_{i}$ after each local epoch and federated communication round t,^38^ see Equation (1).


(1) $$w_{\mathrm{glob}}^{\left(t+1\right)}\;=\;\frac{1}{N_{\mathrm{sites}}}\sum_{i=1}^{N_{\mathrm{sites}}}w_{i}^{\left(t\right)}$$

**Table 1.** Comprehensive overview of benchmarked models in the three distinct evaluation scenarios: personalization, generalization with local training, generalization without local training.

Scenario Models	Personalization	Generalization without local training	Generalization with local training
$L_{i}$	✓	✗	✓
$L_{j\neq i}$	✗	✓	✗
E	✓	✗	✓
$E_{\mathrm{leave}-i-\mathrm{out}}$	✗	✓	✗
FL	✓	✗	✗
${FL}_{\mathrm{leave}-i-\mathrm{out}}$	✗	✓	✓
Spec(E)	✓	✗	✓
Spec(FL)	✓	✗	✗
$\mathrm{Spec}({FL}_{\mathrm{leave}-i-\mathrm{out}})$	✗	✗	✓

The models are local models $L_{i}$, ensemble of those E, federated model FL, and the specializations Spec(E) and Spec(FL) obtained by ensembling E and FL with the local model ($L_{i}$) specific to site i being evaluated.

As the experimental studies serve as proof-of-concept, real-world FL investigations, we chose established DL and FL, neglecting methodological novelty.

#### Evaluation metrics and ranking

To evaluate segmentation performances of trained models, we selected the following metrics according to investigated pathologies.^44–46^ We chose the intersection-based Dice Similarity Coefficient (DSC), as it is the default segmentation metric and suitable for the three target pathologies.^45^^,^^46^ We assessed the segmentation performance using the distance-based metrics Normalized Surface Dice (NSD), suitable for Cons and GGO^45^ with a threshold of 1 mm, and the Hausdorff Surface Distance (HSD), relevant for PE.^46^ The medically relevant difference of predicted and annotated volumes was measured via Normalized Average Volume Error (NAVE).^45^

Based on the utilized metric implementation,^47^ we disregarded samples with an empty ground truth (False Positives). For False Negatives, we set DSC and NSD to 0.0, HSD to 260.0 mm (height of a lung^48^) and NAVE to 20.0, twice the average of True Positives from local models $L_{i}$.

As we consider all metrics as equally relevant, we determine the best performing method through a ranking. We compute for each site and metric the mean metric $\bar{m}$ over all test samples $N_{\mathrm{test}}$ and classes $N_{\mathrm{classes}}$, resulting in $N_{\mathrm{metrics}}$ scores per site. All compared methods are ranked rank(·) per metric score. All $N_{\mathrm{sites}}\times N_{\mathrm{metrics}}$ rankings are averaged to obtain the overall ranking r (Equation (2)).


(2) $$r\;=\;\frac{1}{\;N_{\mathrm{sites}}\;\cdot \;N_{\mathrm{metrics}}}\sum_{i=1}^{N_{\mathrm{sites}}}\sum_{m=1}^{N_{\mathrm{metrics}}}\mathrm{rank}\left(\bar{m}\right)$$

#### Study design

In our experimental studies, we demonstrate for *C2* the execution of real-world FL experiments within our built FL infrastructure. First, we investigate data characteristics via descriptive statistics assessing the data heterogeneity.

Beyond previous studies,^36–38^ we benchmark for *C3* the model performances trained via federated learning (FL) versus locally trained models at site i ($L_{i}$) and an ensemble of these local models (E), obtained by averaging the softmax probabilities of model predictions. Additionally, we assess specialized versions of the FL and E models, Spec(FL) and Spec(E), by ensembling them with the local model ($L_{i}$) specific to site i being evaluated.

We evaluate the compared models across three distinct evaluation scenarios summarized in Table 1.

The *personalization* scenario evaluates how a participating site can obtain improved models from joining collaborative efforts. Given the heterogeneous annotation procedures, we first investigate personalization capabilities in three-sites experiments with homogeneous annotation procedures each, before extending to six-sites experiments. Thereby, we assess segmentation performance by comparing locally trained models ($L_{i}$), an ensemble of locally trained models (E), a federated trained model (FL), and their specialized versions Spec(E) and Spec(FL). Hereinafter, we explore generalization capabilities of models trained across the three sites with manual annotations.

The second scenario explores *generalization without local training*, focusing on sites that cannot train their own models, therefore rely solely on models trained at other sites or through collaborative efforts of those. We benchmark the model performances of all other local models $L_{j\neq i}$, excluding local model $L_{i}$ while testing on site i, against the ensemble of those local models $E_{\mathrm{leave}-i-\mathrm{out}}$ and a federated trained model excluding site i, ${FL}_{\mathrm{leave}-i-\mathrm{out}}$.

The third scenario addresses *generalization with local training*, targeting sites that have local training capabilities but are hesitant to join real-world FL efforts due to its complexities. We compare the performances of local models $L_{i}$, the ensemble of those, E, a federated trained model excluding site i, ${FL}_{\mathrm{leave}-i-\mathrm{out}}$, and the specializations Spec(E) and $\mathrm{Spec}({FL}_{\mathrm{leave}-i-\mathrm{out}})$.

## Results

### Insights in building real-world FL initiatives

For *C1*, we share our experiences from developing a real-world FL initiative, complemented with insights from literature.^4–16^^,^^19–34^ We compiled them into a comprehensive guide (Table 2) and a Gantt chart (Figure A3), navigating through various phases, steps, and issues of translating FL into the real world.

**Table 2.** Detailed guide to build and conduct real-world FL in radiological research outlining the phases, steps, and associated issues.

Steps & Issues (D: difficulty, H: hurdle, S: solution)
**Phase 1: Organization**
Step 1: Identify and convince sites with medically and technically motivated governance stakeholders (★,^6^^,^^16^) - Hurdle 1: Encourage sites to contribute communication/computation overhead, high-quality data (★,^24^^,^^27^) *→Solution 1.1: Assess and value each site’s contribution in terms of data quantity, -quality, -heterogeneity, infrastructure (* ^29^ ^,^ ^49^ *)* *→Solution 1.2: Provide sites incentives, e.g, enhanced performance of FL model, scientific acknowledgement, visibility (*★*)*
Step 2: Conclude non-technical agreements covering health protocols, intellectual property (^19^), scientific acknowledgement (★)
Step 3: Identify responsible IT personnel and radiologists at participating sites (★) - *H3*: Ensure each site has motivated, capable, and available IT staff and radiologists (★,^26^) *→S3: Provide incentives for actual involved persons (IT staff, radiologists), e.g, scientific credentials, visibility (*★,^6^^,^^16^*)*
Step 4: Coordinate harmonized acquisition (prospective data) or curation (retrospective data) of radiological images and annotations (★) - H4: Harmonize terminologies within FL initiative (★,^10^^,^^16^) *→S4: Develop a detailed data specification and a glossary (*★^10^*)*
Step 5: Facilitate communication within FL initiative (★) - H5.1: Ensure regular exchange among directly involved participants (★) *→S5.1: Establish dedicated communication channels such as routine meetings, email, and chat systems (*★*)* - D5.2: Incorporate human input across varying time zones and geographic locations (^25^^,^^26^)
Step 6: Select a FL platform (★) - H6: Reach consensus on a unified FL platform used by all sites (★) *→S6: Define requirements, decide using decision-making tools, e.g, pairwise comparison matrix (*★,^19^^,^^26^^,^^34^*)*
Step 7: Identify DL algorithm and FL methodology (★) - D7: Select state-of-the-art DL and FL methods (★)
Step 8: Identify task to evaluate, e.g, communication efficiency, model performance, security robustness (^20^^,^^21^^,^^30^)
Step 9: Determine best-performing method according to chosen evaluation task (★) - H9: Identify suitable evaluation metrics (★) *→S9.1: Choose evaluation metrics from literature solving related problems (*★*)* *→S9.2: follow advising works, e.g,* ^44^ *for evaluation model performances on medical image data (*★*)*
**Phase 2: Legal requirements**
Step 1: Conclude contracts between FL initiative and participating sites, e.g, for data processing, ethics (★) - H1: Design legal framework across diverse legal jurisdictions (★,^15^) *→S1: Draft individual legal contracts between the FL initiative and each site (*★,^14^*)*
Step 2: Develop legal framework for sharing of DL models within the FL initiative (★) - H2: Determine if DL models constitute patient-related data (★) *→S2: Classify shared DL models as non-patient data (*★*)*
Step 3: Develop legal framework between FL platform provider and participating sites (★) - H3: Define the scope of support that FL platform engineers can provide on-site (★) *→S3: Conclude contracts allowing on-site support of FL platform engineers (*★*)* - D3: Ensure compliance with each site’s software audit requirements (^16^)
**Phase3: Infrastructure set-up**
Step 1: Acquire, secure, and connect hardware resources on-site (★,^5^^,^^16^^,^^20^^,^^21^^,^^25–31^^,^^33^^,^^34^) - D1: Obtain permissions to reserve disk space on limited resources (^4^^,^^9^^,^^16^)
Step 2: Provision and access VMs for deploying the FL platform on-site (★,^16^)
Step 3: Install the FL platform within the clinical IT ecosystem (★) - H3.1: Sites with highly restricted network access (★) *→S3.1: Offer an offline installation option for the FL platform (*★*)* - D3.2: Prevent site-specific customizations of FL platform affecting e.g, functionality of federated communication (★)
Step 4: Configure network settings to allow site VMs to access the container registry and connect to the FL server (★,^16^)
Step 5: Locate data in relevant source systems and configure secure read-access (^16^)
Step 6: Identify and configure communication endpoints between FL platform and third-party systems (★,^16^) - H6: communication issues between FL platform and third-party systems (★) →*S6: Debug communication issues with respective engineers and local IT (*★,^5^^,^^28^*)*
Step 7: Manage urgent features requests or bug fixes in FL platform (★) - D7.1: Required features/hotfixes do not necessarily align with software development cycles (★) - H7.2: Coordinate roll-out of FL platform hot-fix releases (★) *→S7.2: Conduct joint sessions between FL platform engineers and local IT (*★*)* - D7.3: Maintain version compatibility of the FL platform across the initiative (★)
**Phase 4: Experiment preparation**
Step 1: Data mapping and harmonization (★,^10^^,^^11^) - D1.1: Map custom data formats to standard (e.g, FHIR) (^16^) - D1.2: Align differing data formats between the FL platform and third-party systems (★)
Step 2: Data curation (★,^11^) - D2: Balance the ability of FL researchers to retrieve relevant on-site data with data privacy (^19^^,^^26^)
Step 3: Data filtering and ensuring of DL-readiness (★) - H3.1: Address unharmonized, low-quality data and annotations (★,^15^^,^^19^^,^^23^^,^^24^^,^^27^^,^^34^) *→S3.1: Curate data carefully on FL platform by visual or metadata inspection (*★*)* - H3.2: Resolve inconsistencies in data-annotation references (★,^15^) *→S3.2: Employ data validation workflows to verify data-annotation referencing, e.g, via StudyInstanceUID in DICOM (*★*)* - H3.3: Handle data being corrupted or violating standards or specifications (★) *→S3.3: Employ data validation workflows to verify all data attributes are suitable for processing by the DL algorithm, e.g, dimensionality, orientation, number of annotation labels (*★*)*
Step 4: Handling of data-on-algorithm issues (★) - H4.1: DL algorithm fails on site's data (★) *→S4.1: Debug DL algorithm on site’s data with FL researchers and site IT (*★*)* - H4.2: Malicious data identified solely through worse model performances at specific sites (★) *→S4.2: Conduct manual inspection of data to identify invalid samples missed by data validation workflows (*★,^13^*)*
**Phase 5: Experiments and evaluation**
Step 1: Running FL experiments (★) - D1.1: Minimize strain on local hospital resources (^16^) - D1.2: Manage limited communication capacities (^9^) - D1.3: Manage resource availability if FL platform is used in multiple projects (★) - D1.4: Delayed convergence of FL model due to data heterogeneity (^25^)
Step 2: Handling stragglers (★) - D2.1: Varying durations of federated communication rounds due to heterogeneous hardware, network connections (★,^12^^,^^25^^,^^29^^,^^31^^,^^32^) and communication latencies (^25^) - D2.2: Idle machine times at faster sites due to stragglers (★)
Step 3: Managing failing FL experiments (★) - D3.1: Failing FL experiments (^28^^,^^31^^,^^33^) caused by drop-outs of offline sites (★,^6^^,^^25^), IT issues, and configurations like nightly VM backups, GPU driver mismatch (★) - H3.1: Handle failing ((^28^^,^^31^^,^^33^) or offline sites (^25^) resiliently (★) *→S3.1: Save model checkpoints for restarts of failed FL experiments (*★*)* - D3.2: Increased on-site debugging efforts due to error-prone experiments (★), insufficient error logging (★,^13^), limited technical documentation of FL platform (^13^)
Step 4: Restarting failed FL experiments (★) - H4.1: Ensure site’s readiness before restarting a FL experiment, including checking of logs, bugs, resources, and model checkpoints (★) *→S4.1: Automatically share error logs* *→S4.2: Communicate between FL researchers and site IT to resolve bugs, and verify availability of model checkpoints and resources (*★*)*
Step 5: Evaluate model performance on sites (★) - H5: Have final FL model available (★) *→S5.1: FL platform saves final FL model in one additional federated communication round (*★*)* *→S5.2: Compress binaries of final FL model and send via communication channel, e.g, zip-file via mail (*★*)*
Step 6: Handling issues of test data on evaluation algorithm (★) - D6.1: Test data filtering and ensuring of DL-readiness via data validation workflows (★) - H6.2: Evaluation algorithm fails on site's test data (★) *→S6.2: Debug evaluation algorithm on site’s data to resolve issue with FL researchers and site IT (*★*)*

Literature insights are cited; our insights are marked with ★.

Practical hurdles in organizing a FL initiative are convincing site’s IT departments and governance stakeholders to participate through incentives,^6^^,^^16^ and the importance of harmonizing terminology across the initiative.^16^ Organizational issues to consider are methods to assess and value sites’ data quantity, quality, heterogeneity, and infrastructure contributions,^29^ the need for available on-site personnel (IT staff, expert annotators),^26^ and strategies for incorporating human oversight, particularly across time zones.^25^^,^^26^ Establishing clear governance, traceability, and accountability for human expertise and site policies,^26^ and setting technical requirements for the FL platform regarding data access history, training configurations, and error handling should be clarified.^19^^,^^34^ Further practical hurdles include the need to create detailed specifications for radiological imaging data and annotations,^10^ establishing effective communication channels like regular meetings and chat rooms among FL researchers, radiologists, and IT staff, and aligning the FL platform's development cycle with necessary features and fixes. Selecting the utilized state-of-the-art DL and FL method are difficulties to clarify.

Legal steps involve negotiating bidirectional contracts between sites and the FL initiative,^14^ potentially across diverse legal jurisdictions,^15^ addressing regulations regarding software support and audits,^16^ and agreements considering model weights as non-patient-related, shareable data.

Practical steps in infrastructure setup include essential on-site infrastructure requirements,^5^^,^^20^^,^^21^^,^^25–31^^,^^33^^,^^34^ acquiring and connecting hardware, provisioning and accessing on-site virtual machines (VMs),^16^ managing limited disk space and resources,^5^^,^^9^^,^^16^ configuring firewall permissions, and establishing communication with third-party systems, e.g, PACS and annotation tools.^5^^,^^16^^,^^28^ Technical difficulties include minimizing the strain on site resources^16^ and managing limited communication capacities.^9^ Further significant practical issues involve installing FL platforms in highly restricted clinical IT environments and debugging sessions between FL platform engineers and local IT resolving network access and communication issues between the FL platform and third-party systems.

During the experiment preparation phase, practical hurdles primarily involve data-related issues including low quality but high heterogeneity,^15^^,^^19^^,^^23^^,^^24^^,^^26^^,^^27^^,^^29^^,^^34^ the necessity to standardize custom data,^16^ and addressing missing data harmonization or inconsistencies.^11^^,^^15^ Additionally, insufficient data specifications leading to preventable data heterogeneity,^13^ and data handling that violates standards or specifications. Ensuring data readiness for experiments can be achieved by deploying automated data validators, which minimize the need for manual inspections by local IT staff, and by FL researchers regularly conducting sanity checks on experimental results. Additionally, the necessity for on-site debugging possibilities for FL researchers^29^ and the trade-off between allowing researchers to find relevant data on-site while maintaining data privacy,^19^^,^^26^ represent considerable issues.

In the phase of experiments and evaluation, hurdles include managing infrastructure failures, such as site dropouts^6^ due to straggling,^25^ crashing,^28^^,^^31^^,^^33^ and disconnected sites,^27^ which require experiment restarts leading to idle machines at other sites. These issues are exacerbated by insufficient error logging and limited technical documentation, necessitating on-site debugging.^13^ Technical difficulties involve time variations per federated communication round due to infrastructure heterogeneity across the initiative,^12^ the necessity to minimize strain on site’s resources,^16^ managing limited communication capacities^9^ and dealing with data heterogeneity delaying the convergence of federated models.^25^ Lastly, it is crucial to ensure that the final FL model is readily available on-site for evaluation.

### Real-world FL study: experimental results

For *C2*, the experimental results demonstrate the functionality of our FL infrastructure, built and deployed by successfully overcoming the previously identified practical hurdles and difficulties, underscoring the practical relevance of our proposed guide. We benchmarked benefits hospitals gain by leveraging the power of FL, demonstrated across three distinct evaluation scenarios for *C3*.

#### Data characteristics

Data heterogeneity across participating sites impacts performance of models trained via FL.^50^ We examined the characteristics of the distributed data to understand the degree of heterogeneity (descriptive statistics in Figure 4). Since CTs were provided pseudonymized, we relied on technical metadata and image-derived characteristics, excluding demographic details of the cohort.

The dataset comprises CT scans from four manufacturers, predominantly Siemens and Philips, with site CHA being an outlier. The voxel volumes vary from 0.15 (site UKK) to 4.84 mm^3^ (site UME). The HU intensity distributions across the scans are similar due to normalization of HU values. Annotation labels, including PE as the least occurring pathology, are uniformly distributed across sites. We analyzed the distinctions between site’s annotations by examining annotation volumes and conducting a connected component analysis (CCA). Site CHA features the largest annotation volumes, especially for PE cases. The CCA highlighted significant variations in annotation procedures among sites, with automatically pre-processed sites (CHA, UKK, UKKI) having a higher count of connected components (CC) compared to manually annotated sites (TUM, UME, UKF) (Figure A1).

### Segmentation performance evaluation

#### Personalization

**Figure 5.**
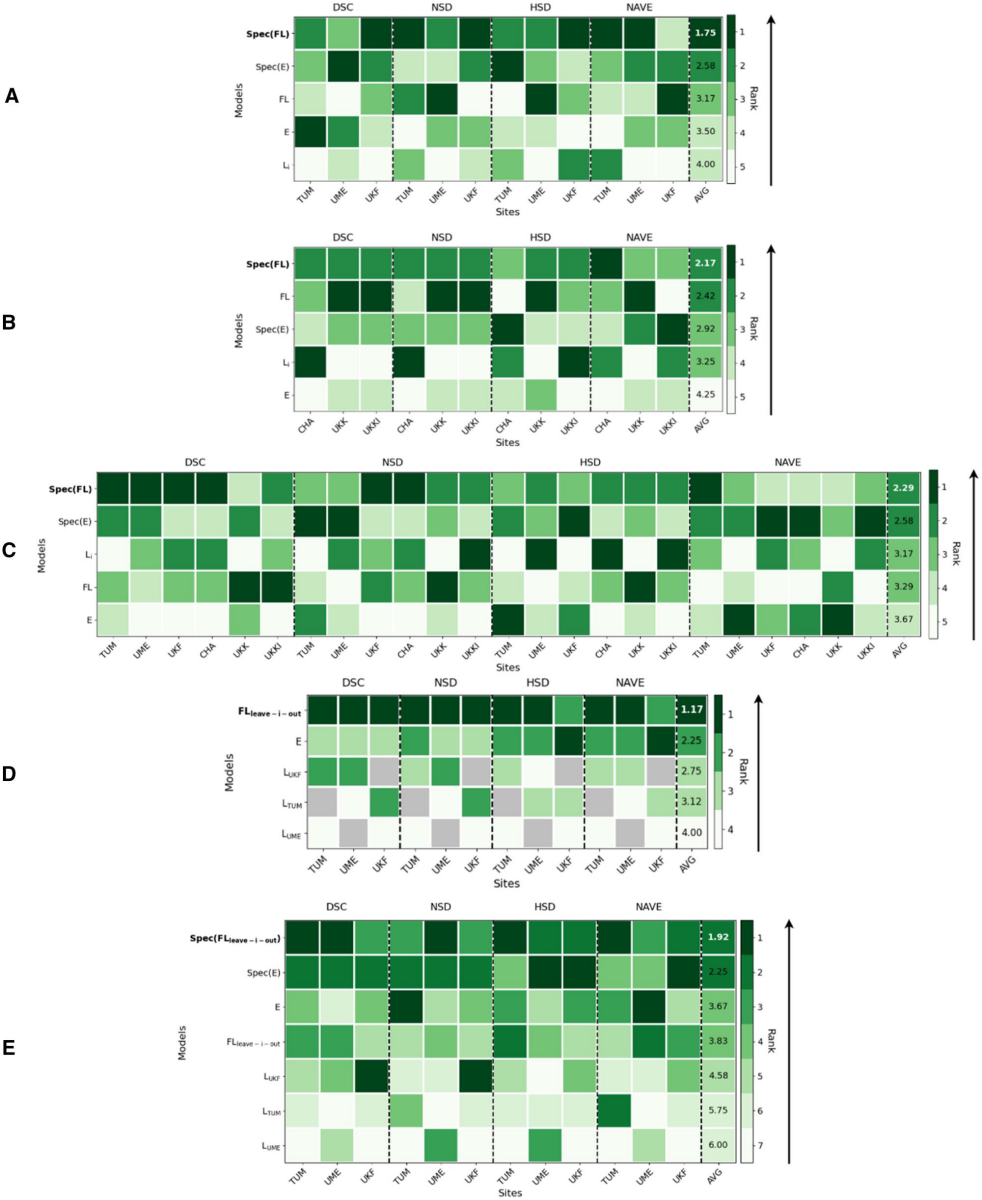
Heatmap visualization of the ranks achieved by compared models in the five evaluation scenarios (A-E) over the four metrics (DSC, NSD, HSD, NAVE). Each row represents a model, with darker squares indicating better performance and ranks. The final column shows the average rank for each model, with the arrow pointing towards the best rank. The evaluation scenarios are (A) Personalization of manually annotated sites; (B) Personalization of automatically pre-processed sites; (C) Personalization of all sites; (D) Generalization without local training of manually annotated sites; (E) Generalization with local training of manually annotated sites.

**Figure 6.**
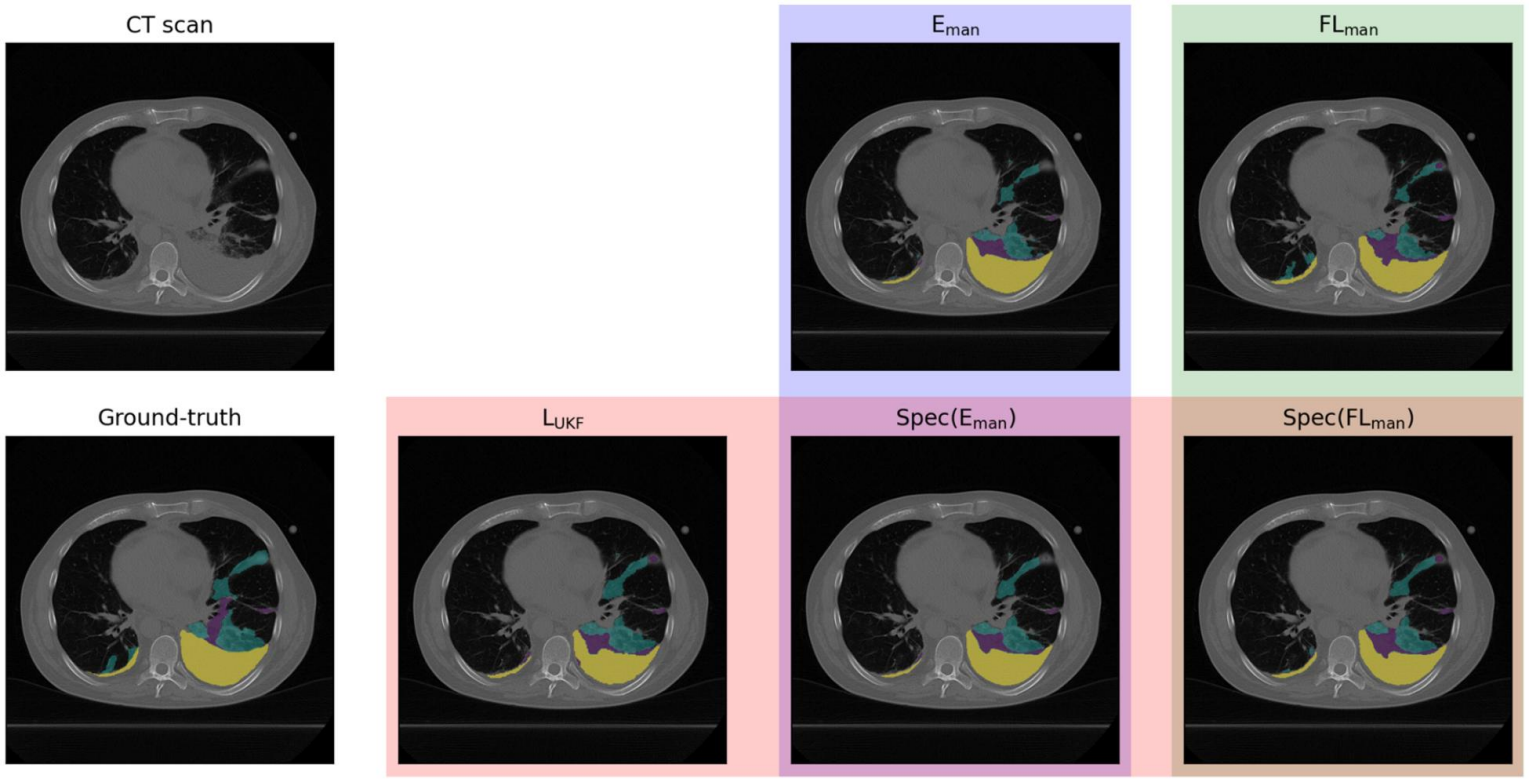
Qualitative segmentation results on a test sample from site UKF with Cons in violet, GGO in cyan, PE in yellow. Segmentation predictions of the models $L_{\mathrm{UKF}}$, $E_{\mathrm{man}}$, ${FL}_{\mathrm{man}}$, with the specialization approach for $\mathrm{Spec}(E_{\mathrm{man}})$ and $\mathrm{Spec}({FL}_{\mathrm{man}})$ highlighted.

Evaluating personalization capabilities among manually annotated sites (TUM, UME, UKF) shows an overall superior performance of $\mathrm{Spec}({FL}_{\mathrm{man}})$ achieving the best rank with average metrics: DSC = 0.47 (95% CI: 0.43-0.52), NSD = 0.39 (95% CI: 0.35-0.44), HSD = 127.94 mm (95% CI: 114.40-141.57) and NAVE = 6.20 (95% CI: 0.0-13.57). The introduced specialization stabilizes the segmentation performances across all metrics, whereas non-specialized models ($L_{i}$, $E_{\mathrm{man}}$, ${FL}_{\mathrm{man}}$) show considerable variability. We conclude that collaborative approaches outperform local models $L_{i}$, while among non-specialized collaborative approaches ${FL}_{\mathrm{man}}$ outperforms $E_{\mathrm{man}}$ (Figure 5A, Table A2, Figure A4; qualitative results in Figure 6).

Considering personalization capabilities across automatically pre-processed sites (CHA, UKK, UKKI), we obtain superior performance of the specialized and non-specialized FL models ($\mathrm{Spec}({FL}_{\mathrm{auto}})$ and ${FL}_{\mathrm{auto}}$) on average rank with average metrics for $\mathrm{Spec}({FL}_{\mathrm{auto}})$ of DSC = 0.41 (95% CI: 0.40-0.51), NSD = 0.40 (95% CI: 0.39-0.49), HSD = 127.96 mm (95% CI: 106.51-134.51), NAVE = 2.02 (95% CI: 0.49-3.23). Despite $\mathrm{Spec}({FL}_{\mathrm{auto}})$ achieving only a single first-place ranking, its specialization contributes to a more consistent performance across metrics, securing its top position on average rank. Moreover, the results reveal a trend that sites with poor local model performance gain greater benefits from FL (Figure 5B, Table A3, Figure A5).

Incorporating all six sites in the benchmarking introduces a high data heterogeneity due to differences in annotations. Despite this, we obtain for $\mathrm{Spec}({FL}_{\mathrm{all}})$ the best average ranking with average metrics of DSC = 0.44 (95% CI: 0.43-0.50), NSD = 0.38 (95% CI: 0.36-0.43), HSD = 136.48 mm (95% CI: 122.60-143.33) and NAVE = 10.10 (95% CI: 0.0-35.81). The results support previously observed trends that specialization brings more stable performances and ranks, while sites with poorer local performance notably benefit from FL (Figure 5C, Table A4, Figure A6). Additionally, it can be observed that expanding an FL initiative does not necessarily lead to improved model performance (Tables A2, A3, and A5).

#### Generalization without local training

The generalization performance is evaluated among the three manually annotated sites TUM, UME and UKF. The best generalizing model on average rank is ${FL}_{\mathrm{leave}-i-\mathrm{out}}$ with average metrics of DSC = 0.42 (95% CI: 0.37-0.46), NSD = 0.33 (95% CI: 0.29-0.37), HSD = 140.93 mm (95% CI: 127.30-155.77) and NAVE = 9.52 (95% CI: 0.0-23.97). The results reveal that local models $L_{i\neq j}$ from other sites j do not generalize well, whereas collaborative approaches, particularly with ${FL}_{\mathrm{leave}-i-\mathrm{out}}$, demonstrate superior generalization capabilities (Figure 5D, Table A6, Figure A7).

#### Generalization with local training

For sites with training capabilities seeking to circumvent efforts of real-world FL, the results consistently reveal that collaborative approaches are superior compared to local models $L_{i}$. Although non-specialized E outperforms ${FL}_{\mathrm{leave}-i-\mathrm{out}}$, the top-performing model is ${\mathrm{Spec}(FL}_{\mathrm{leave}-i-\mathrm{out}})$ with average metrics of DSC = 0.46 (95% CI: 0.41-0.50), NSD = 0.37 (95% CI: 0.33-0.41), HSD = 125.48 mm (95% CI: 112.06-139.92), NAVE = 3.65 (95% CI: 1.20-6.08). This suggests that the greatest benefit for site i is achieved by adopting a FL model trained by other sites j and specializing it with their local model $L_{i}$ (Figure 5E, Table A7, Figure A8).

## Discussion

The guide we propose for building real-world FL initiatives (*C1*) details steps, identifies hurdles, and provides solutions that we have implemented, steering clear of hypothetical solutions. All issues associated with real-world FL were either resolved or circumvented, whereas key solutions included ensuring that each site had motivated and capable staff through targeted incentives such as scientific acknowledgement. Additionally, defining data specifications during the organizational phase and employing data validators in the experiment preparation phase were crucial to ensure data-readiness. Insufficient data specifications led to varied annotation procedures among radiologists, resulting in poorer model performance (Figure 5B). This was evident from the performance disparities observed between sites with manual versus automatically pre-processed annotations (Tables A4-A6). Our most effective solution involved providing offline-installable VMs to deploy FL platforms at sites with restricted internet access. Conversely, our least effective solution was lengthy troubleshooting sessions via video conferences between FL platform-, third-party system engineers and on-site IT staff debugging software interfaces or deployed algorithms on site’s data instead of accessing the server remotely. Significantly hindering difficulties were idle machines at faster sites due to heterogeneous infrastructure and frequent errors during the initial experimental phase until we identified and addressed contributing factors like nightly backups and other maintenance activities conducted by local IT.

Our experimental studies demonstrate the functionality of our FL infrastructure, emphasize the relevance of our proposed guide for real-world applications (*C2*) and address *P3* by benchmarking FL against alternative approaches to determine if it is worth the inherent challenges (*C3*). First, we analyzed data characteristics assessing the real-world data heterogeneity. This proved invaluable, as it enabled to detect differences in annotation procedures using a CCA, highlighting a significant source of data heterogeneity. Regarding segmentation performances, collaborative approaches—E, Spec(E), FL, and Spec(FL)—consistently outperformed local models $L_{i}$ in all evaluation scenarios, highlighting the power of collaborative training approaches. Among these, the FL model and the specialized Spec(FL) showed superior performance over ensembling approaches in both personalization and generalization scenarios. Moreover, our experiments demonstrate that randomly adding sites to a federation can worsen model performances due to increased data heterogeneity (Tables A2, A3, and A5). Our results address research problem *P3* that despite the substantial hurdles involved in conducting real-world FL, the benefits clearly justify the investment. By jointly considering these hurdles and showcasing FL's superiority, we provide the complete picture regarding real-world FL in radiology.

The proposed guide serves as a starting point, recognizing that it won't address all potential issues and is intended for extension by future real-world FL efforts. While we supplemented our experiences with relevant FL literature, some insights may be unique to our initiative and not universally applicable. Our achieved performances on the challenging segmentation task^45^^,^^51^ are comparable to those reported in studies deploying non-fine-tuned segmentation models investigating the same target pathologies.^51–53^ The results of our proof-of-concept experiments are influenced by the following factors: data heterogeneity from varying annotation procedures, choosing the default federated averaging aggregation strategy, and using a single fold for nnU-Net rather than five-fold cross-validation without further hyper-parameter optimization. Additionally, results were influenced by efficiency-driven decisions, as using nnU-Net’s low-resolution model or training for fixed 1000 epochs, the inclusion of sites with low segmentation performances on own test data, and the lack of assessing annotation inter-rater variability.

Despite its limitations, our study outlines possibilities for future research. With the established FL infrastructure in the RACOON initiative, we are now equipped to investigate clinically relevant research questions at scale, leveraging the power of FL. From a FL perspective, exploring selection strategies for participating sites emerges as a promising area of research, particularly given the observed impact of heterogeneous annotations on model performance. This could lead to development of automatic proxies pre-evaluating a site’s participation in FL training. Moreover, respecting the lower effort and strong performance of ensemble approaches compared to local models, further explorations how ensemble approaches can enhance personalization and generalization are advantageous.

## Conclusion

In this work, we strive towards bridging the gap between simulated and real-world FL research. We identified significant gaps in literature: the lack of detailed, expertise-proven insights into establishing real-world FL initiatives (*P1* and *P2*), and the absence of extensive benchmarking of FL against alternatives in real-world settings justifying its adoption despite inherent challenges (*P3*). To address this, we developed and deployed a FL initiative within the German RACOON project and compiled our insights into a comprehensive guide (*C1*). This guide details necessary steps, encountered issues and suggests solutions involved in building and deploying real-world FL in radiological research. We conducted real-world experiments validating the functionality of our infrastructure, underscoring the practical relevance of our proposed guide (*C2*), and demonstrating FL’s superiority among collaborative learning approaches, proving its value despite hurdles in real-world FL (*C3*). With these three contributions, we provide an all-encompassing consideration of real-world FL and aim to streamline future real-world FL initiatives by guiding through the development process and helping avoiding pitfalls. We target to advance FL's clinical adoption, enhancing diagnosis and therapy with collaboratively trained models on distributed data.

## Supplementary Material

ocae259_Supplementary_Data

## Data Availability

The radiological image data used in this study is private, sensitive, and owned by the participating hospitals. Due to privacy regulations and institutional policies, this data cannot be shared publicly. However, the open-source code for the FL platform Kaapana including all implementations used for the experimental studies are openly available at https://github.com/kaapana/kaapana.
