# Supplementary material for: Real-world federated learning in radiology: hurdles to overcome and benefits to gain
Source: J Am Med Inform Assoc. 2024 Oct 25;32(1):193–205. doi: 10.1093/jamia/ocae259 (PMC11648732; doi:10.1093/jamia/ocae259)
Supplement: ocae259_Supplementary_Data [file ocae259_supplementary_data.docx]

# Supplementary Materials

|  | Cons | GGO |
| --- | --- | --- |
| man  (UKF) | 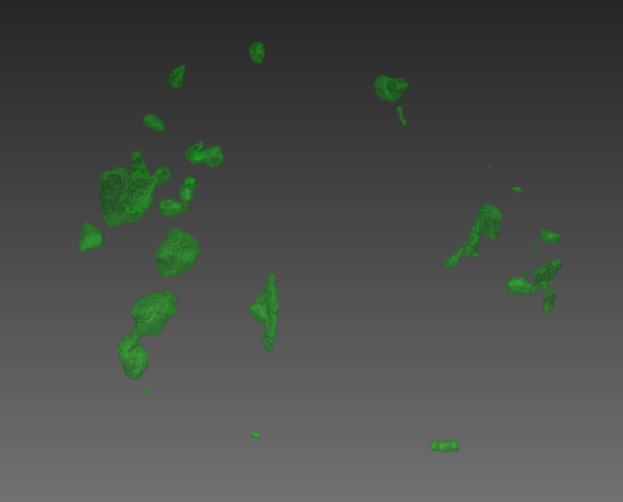 | 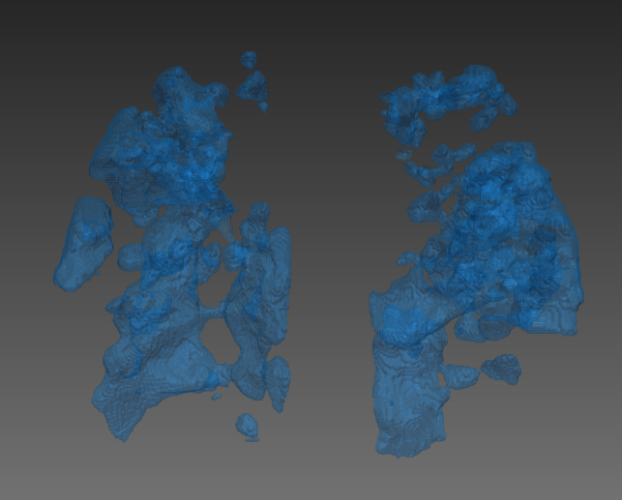 |
| auto  (UKKI) | 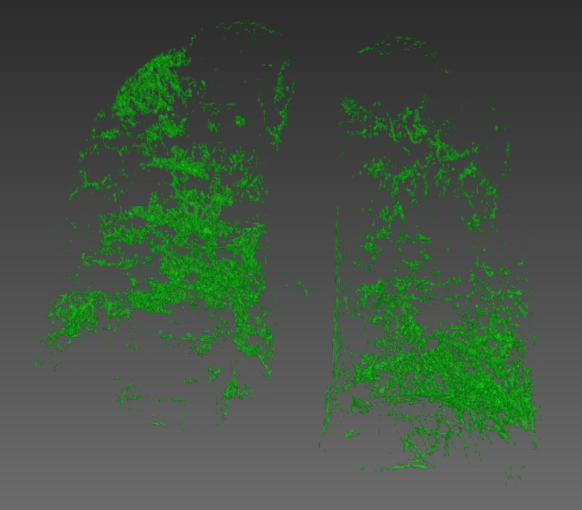 | 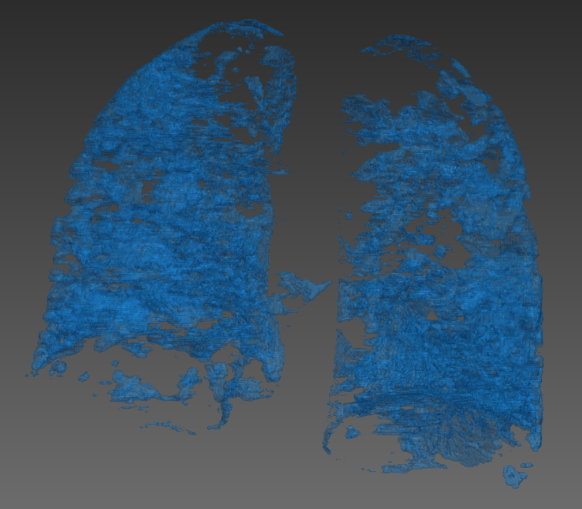 |

Figure A.1: Qualitative comparison of a manually generated (man), and an automatically preprocessed (auto) annotation mask. Both annotation masks visualize Cons in green, GGO in blue, PE is not present. Annotation masks are from sites UKF and UKKI, respectively, and illustrate different patients.


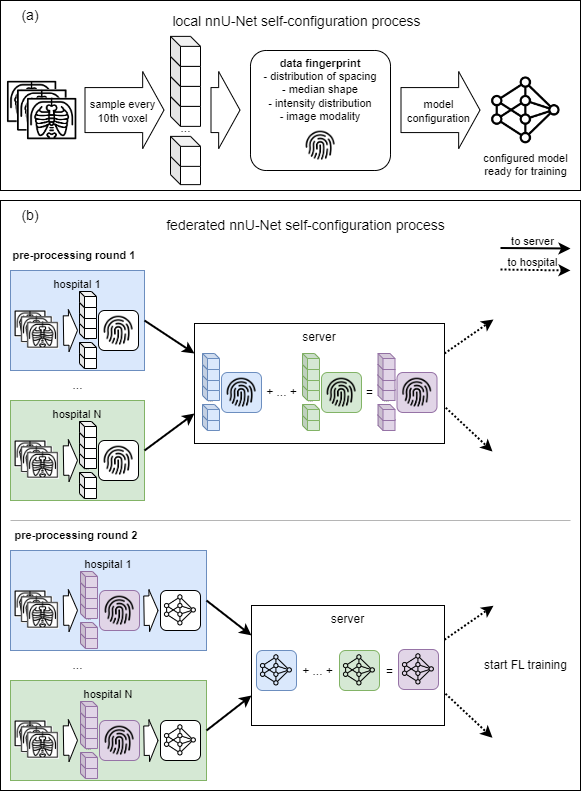


Figure A.2: Local (a) and federated (b) self-configuration of the nnU-Net segmentation model. For the local self-configuration, a dataset fingerprint is generated from the local training data and a corresponding model is configured. The federated self-configuration is divided into two pre-processing rounds: First, each site generates the local data fingerprints which are shared and aggregated by the server. Second, each site configures and initializes from the aggregated data fingerprint a model, that is shared and aggregated by the server. Finally, the aggregated model is shared with the sites to start the FL training process.

Table A.1: nnU-Net parameters of models FL_man_, FL_auto_ and FL_all_.

| **model** | **FL_man_** | | **FL_auto_** | | **FL_all_** | |
| --- | --- | --- | --- | --- | --- | --- |
| **parameters** | **stage 0** | **stage 1** | **stage 0** | **stage 1** | **stage 0** | **stage 1** |
| **Batch size** | 2 | 2 | 2 | 2 | 2 | 2 |
| **Number of pooling per axis** | [4, 5, 5] | [4, 5, 5] | [4, 5, 5] | [4, 5, 5] | [4, 5, 5] | [4, 5, 5] |
| **Patch size** | [112, 160, 128] | [112, 160, 128] | [96, 160, 160] | [96, 160, 160] | [112, 160, 128] | [112, 160, 128] |
| **Median image shape in voxels** | [165, 233, 233] | [361, 512, 512] | [157, 250, 250] | [322, 512, 512] | [164, 233, 233] | [164, 233, 233] |
| **Original spacing [mm]** | [0.90, 0.77, 0.77] | [0.90, 0.77, 0.77] | [1.00, 0.72, 0.72] | [1.00, 0.72, 0.72] | [0.90, 0.75, 0.75] | [0.90, 0.75, 0.75] |
| **Target spacing [mm]** | [1.98, 1.70, 1.70] | [0.90, 0.77, 0.77] | [2.05, 1.47, 1.47] | [1.00, 0.72, 0.72] | [1.98, 1.64, 1.64] | [0.90, 0.75, 0.75] |
| **Pooling operation kernel sizes** | [[2, 2, 2], [2, 2, 2], [2, 2, 2], [2, 2, 2], [1, 2, 2]] | [[2, 2, 2], [2, 2, 2], [2, 2, 2], [2, 2, 2], [1, 2, 2]] | [[2, 2, 2], [2, 2, 2], [2, 2, 2], [2, 2, 2], [1, 2, 2]] | [[2, 2, 2], [2, 2, 2], [2, 2, 2], [2, 2, 2], [1, 2, 2]] | [[2, 2, 2], [2, 2, 2], [2, 2, 2], [2, 2, 2], [1, 2, 2]] | [[2, 2, 2], [2, 2, 2], [2, 2, 2], [2, 2, 2], [1, 2, 2]] |
| **Convolution operation kernel sizes** | [[3, 3, 3], [3, 3, 3], [3, 3, 3], [3, 3, 3], [3, 3, 3], [3, 3, 3]] | [[3, 3, 3], [3, 3, 3], [3, 3, 3], [3, 3, 3], [3, 3, 3], [3, 3, 3]] | [[3, 3, 3], [3, 3, 3], [3, 3, 3], [3, 3, 3], [3, 3, 3], [3, 3, 3]] | [[3, 3, 3], [3, 3, 3], [3, 3, 3], [3, 3, 3], [3, 3, 3], [3, 3, 3]] | [[3, 3, 3], [3, 3, 3], [3, 3, 3], [3, 3, 3], [3, 3, 3], [3, 3, 3]] | [[3, 3, 3], [3, 3, 3], [3, 3, 3], [3, 3, 3], [3, 3, 3], [3, 3, 3]] |


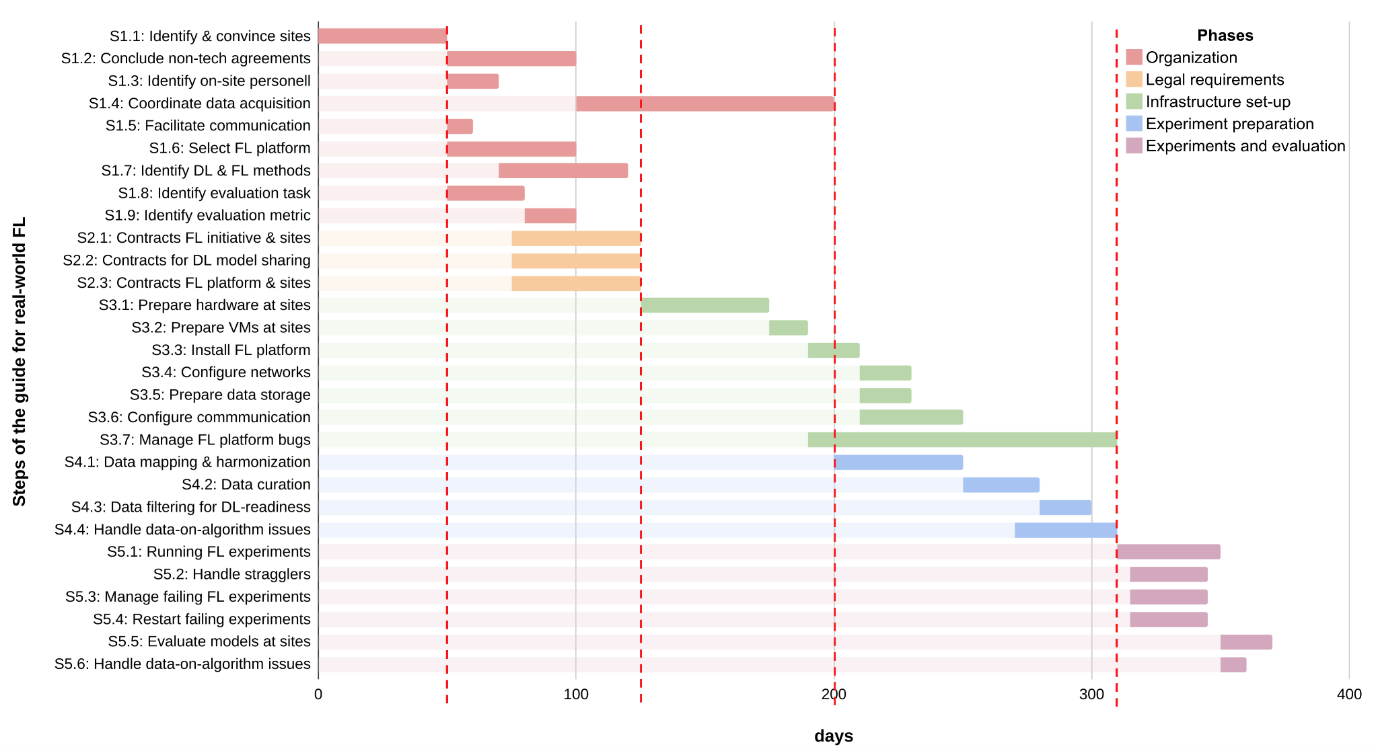


Figure A.3: Gantt chart to the proposed guide to build and deploy real-world FL in radiological research. The steps and phases correspond to those presented in the guide (table 2); we emphasize temporal dependencies of steps neglecting time indications as these are project specific. The red, dotted line indicate four major milestones marking critical requirements for subsequent steps; the milestones are: Identification and convincing sites to participate (step 1.1), Completion of legal requirements (steps 2.1-2.3), Coordination of harmonized data acquisition (step 1.4), Finalization of all preparations to start the real-world FL experiments (steps 3.7 and 4.4).

Table A.2: Personalization segmentation evaluation performance among sites with manually generated annotations (TUM, UME, UKF).

|  | **test data** | | | | | | | | | | | | **avg** | | | | **rank** |
| --- | --- | --- | --- | --- | --- | --- | --- | --- | --- | --- | --- | --- | --- | --- | --- | --- | --- |
|  | **TUM** | | | | **UME** | | | | **UKF** | | | |  |  |  |  |  |
|  | **DSC** | **NSD** | **HSD** | **NAVE** | **DSC** | **NSD** | **HSD** | **NAVE** | **DSC** | **NSD** | **HSD** | **NAVE** | **DSC** | **NSD** | **HSD** | **NAVE** |  |
| **L_i_** | 0,44 | 0,33 | 156,53 | 2,04 | 0,41 | 0,36 | 117,99 | 8,56 | 0,50 | 0,40 | 146,48 | 43,69 | 0,45 | 0,36 | 140,33 | 18,09 | 4,00 |
| **E** | 0,47 | 0,34 | 147,47 | 2,71 | 0,43 | 0,37 | 119,23 | 8,53 | 0,43 | 0,36 | 131,35 | 3,65 | 0,44 | 0,36 | 132,69 | 4,96 | 3,50 |
| **FL** | 0,46 | 0,32 | 146,64 | 1,82 | 0,44 | 0,35 | 155,62 | 4,20 | 0,49 | 0,39 | 133,18 | 2,32 | 0,46 | 0,36 | 145,14 | **2,78** | 3,17 |
| **Spec(E)** | 0,47 | 0,34 | 144,50 | 2,60 | 0,42 | 0,38 | 104,75 | 8,43 | 0,46 | 0,39 | 123,96 | 3,06 | 0,45 | 0,37 | **124,41** | 4,70 | 2,58 |
| **Spec(FL)** | 0,47 | 0,33 | 144,33 | 1,36 | 0,43 | 0,39 | 117,85 | 6,56 | 0,51 | 0,45 | 121,65 | 10,66 | **0,47** | **0,39** | 127,94 | 6,20 | **1,75** |


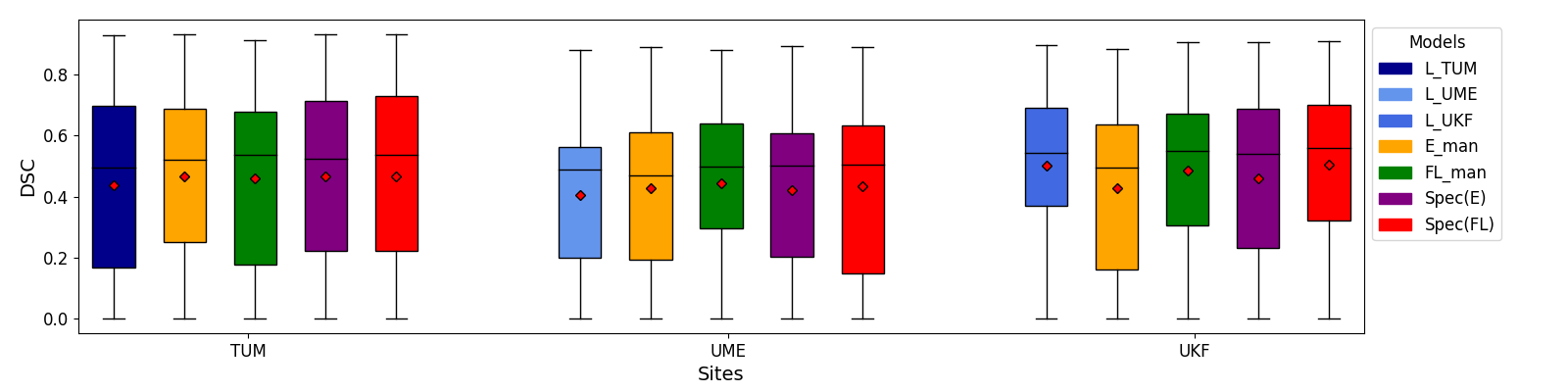


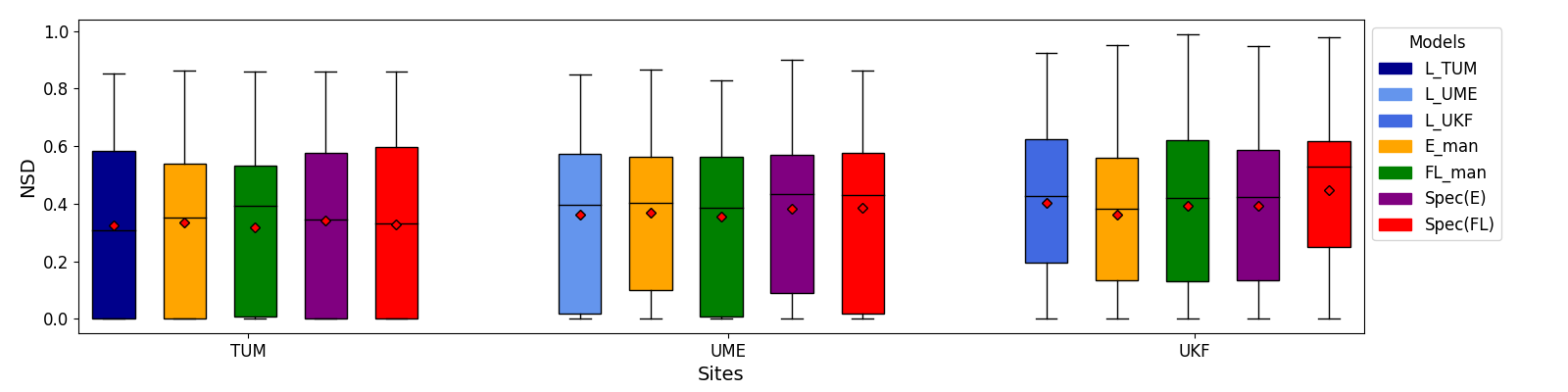


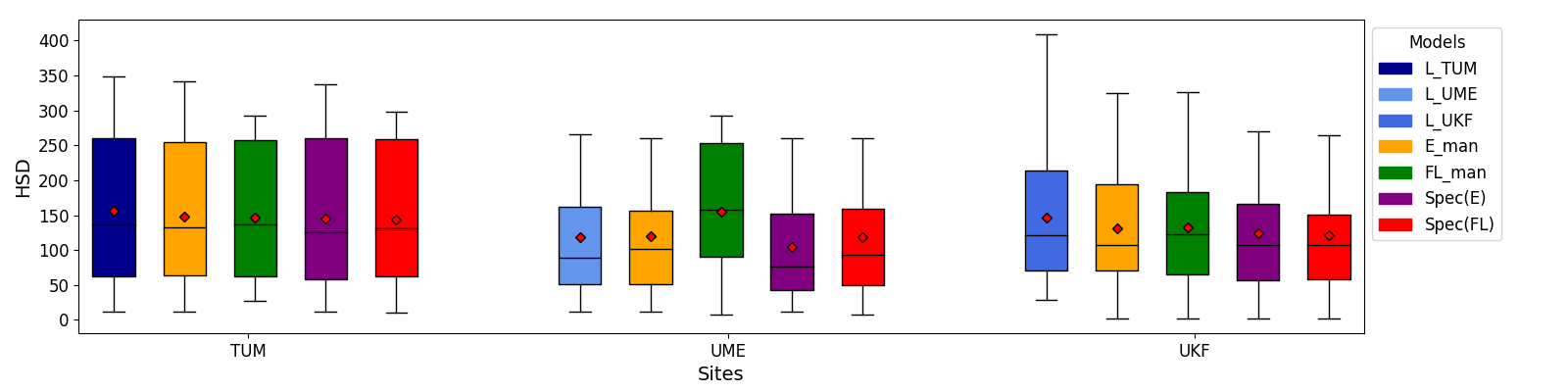


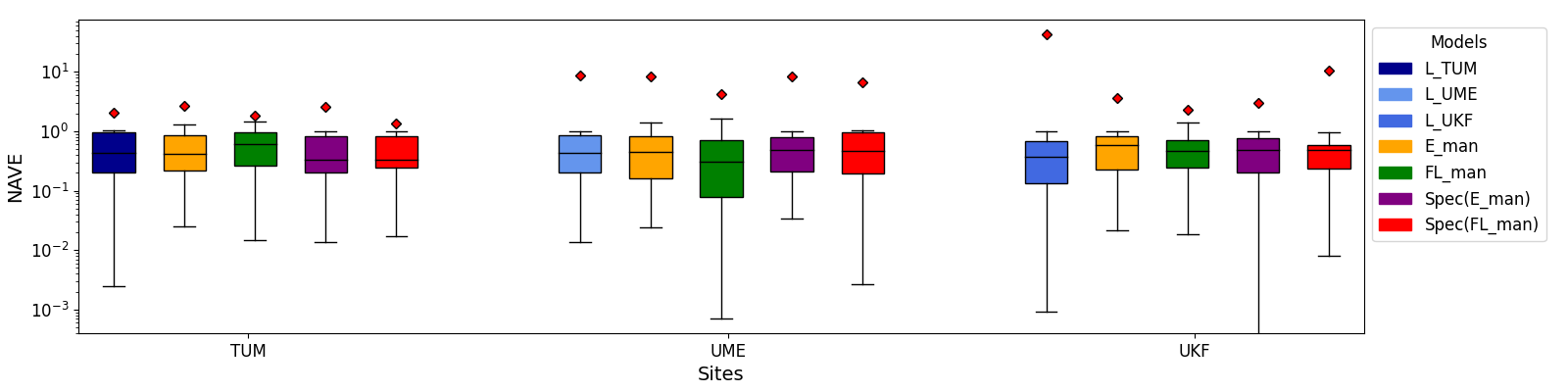


Figure A.4: Boxplots of personalization segmentation evaluation performance among sites with manually generated annotations (TUM, UME, UKF) of metrics DSC, NSD, HSD and NAVE.

Table A.3: Personalization segmentation evaluation performance among sites with automatically pre-processed annotations (CHA, UKK, UKKI).

|  | **test data** | | | | | | | | | | | | avg | | | | **rank** |
| --- | --- | --- | --- | --- | --- | --- | --- | --- | --- | --- | --- | --- | --- | --- | --- | --- | --- |
|  | **CHA** | | | | **UKK** | | | | **UKKI** | | | |  |  |  |  |  |
|  | **DSC** | **NSD** | **HSD** | **NAVE** | **DSC** | **NSD** | **HSD** | **NAVE** | **DSC** | **NSD** | **HSD** | **NAVE** | **DSC** | NSD | HSD | NAVE |  |
| **L_i_** | 0,60 | 0,57 | 100,20 | 1,54 | 0,08 | 0,07 | 214,80 | 4,91 | 0,36 | 0,37 | 115,25 | 1,84 | 0,34 | 0,34 | 143,42 | 2,76 | 3,08 |
| **E** | 0,46 | 0,49 | 107,17 | 2,65 | 0,24 | 0,20 | 176,90 | 2,70 | 0,36 | 0,36 | 135,85 | 2,15 | 0,35 | 0,35 | 139,97 | 2,50 | 4,25 |
| **FL** | 0,55 | 0,52 | 111,97 | 1,62 | 0,39 | 0,34 | 139,86 | 0,49 | 0,38 | 0,35 | 125,92 | 3,66 | **0,44** | 0,40 | 125,92 | 1,92 | 2,75 |
| **Spec(E)** | 0,53 | 0,53 | 95,39 | 1,68 | 0,26 | 0,24 | 182,74 | 2,29 | 0,36 | 0,37 | 127,70 | 1,79 | 0,39 | 0,38 | 135,28 | 1,92 | 2,83 |
| **Spec(FL)** | 0,59 | 0,56 | 101,44 | 1,45 | 0,27 | 0,27 | 163,04 | 2,68 | 0,37 | 0,37 | 119,41 | 1,92 | 0,41 | 0,40 | 127,96 | 2,02 | **2,08** |


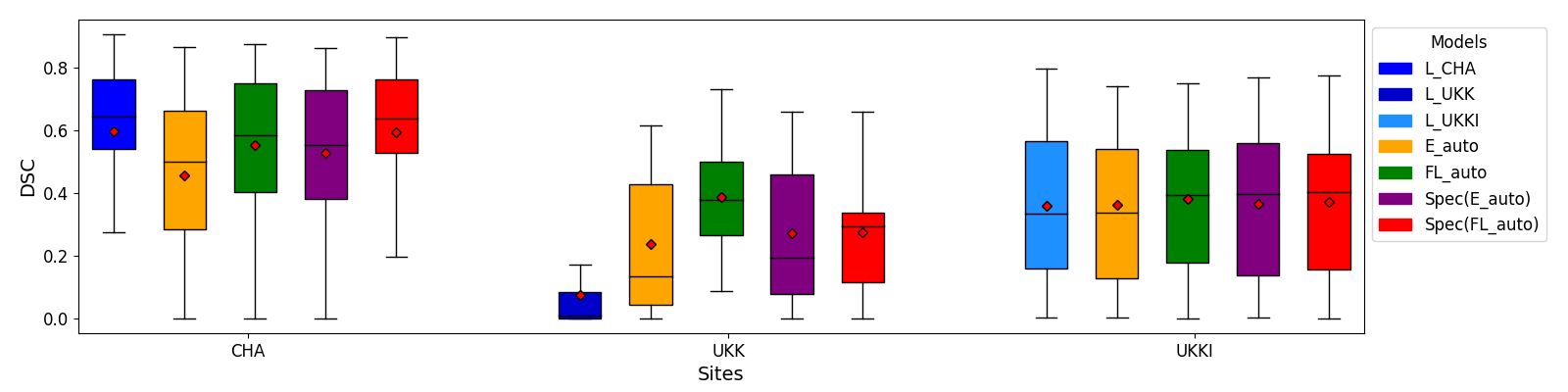


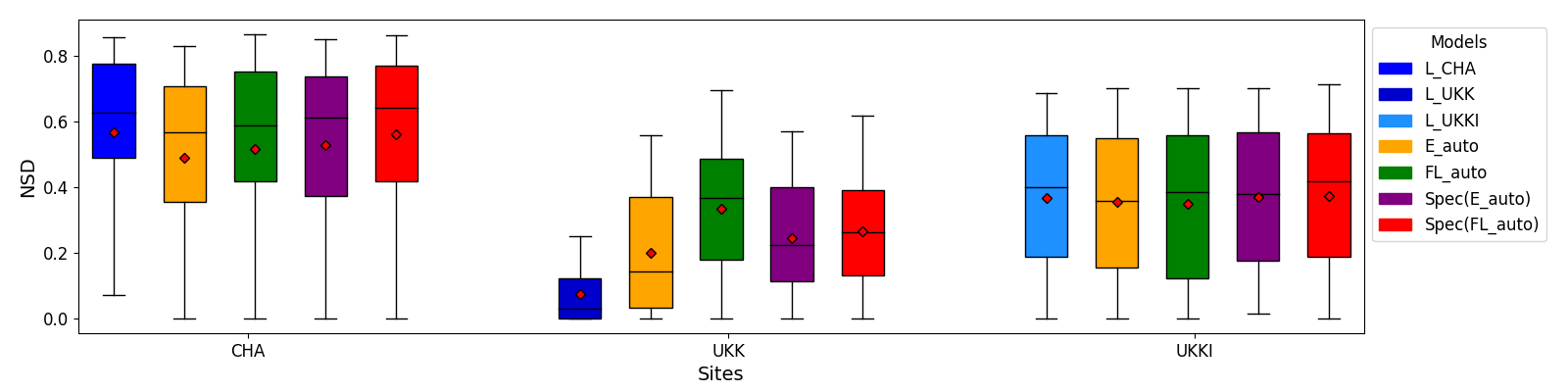

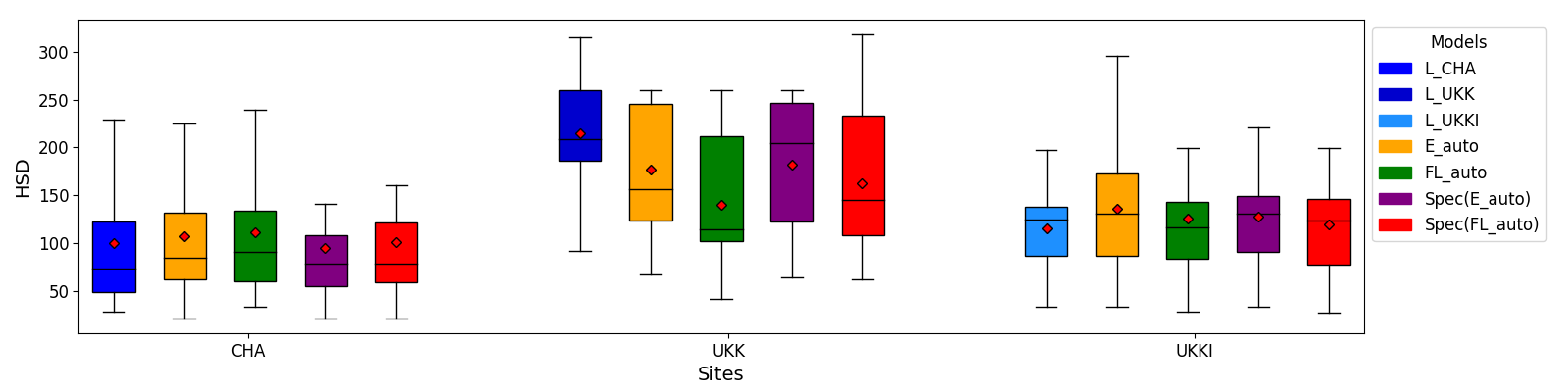

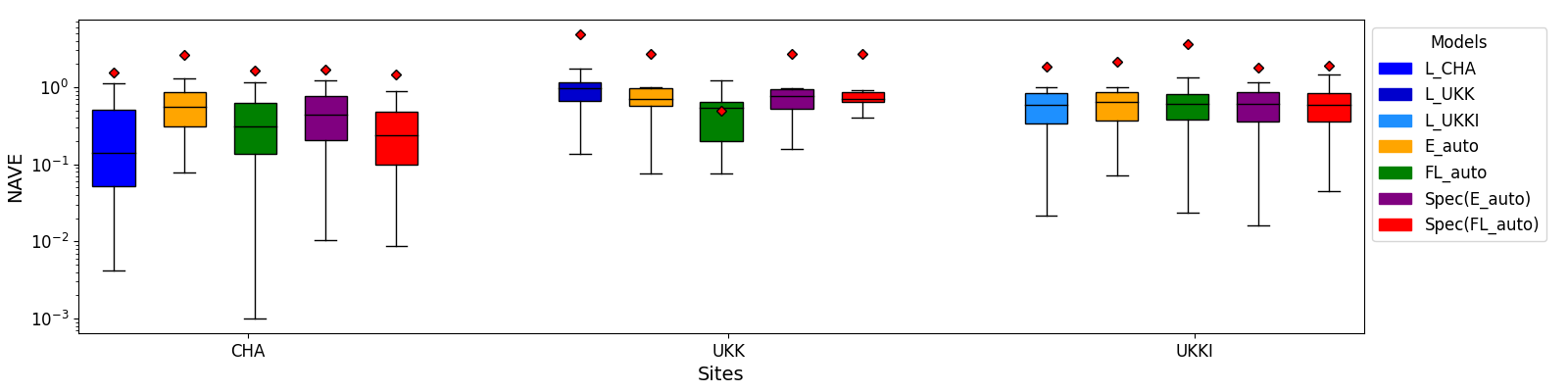


Figure A.5: Boxplots of personalization segmentation evaluation performance among sites with automatically pre-processed annotations (CHA, UKK, UKKI) of metrics DSC, NSD, HSD and NAVE.

Table A.4: Personalization segmentation evaluation performance among all sites.

|  | **test data** | | | | | | | | | | | | | | | | | | | | | | | | **avg** | | | | **rank** |
| --- | --- | --- | --- | --- | --- | --- | --- | --- | --- | --- | --- | --- | --- | --- | --- | --- | --- | --- | --- | --- | --- | --- | --- | --- | --- | --- | --- | --- | --- |
|  | **TUM** | | | | **UME** | | | | **UKF** | | | | **CHA** | | | | **UKK** | | | | **UKKI** | | | |  |  |  |  |  |
|  | **DSC** | **NSD** | **HSD** | **NAVE** | **DSC** | **NSD** | **HSD** | **NAVE** | **DSC** | **NSD** | **HSD** | **NAVE** | **DSC** | **NSD** | **HSD** | **NAVE** | **DSC** | **NSD** | **HSD** | **NAVE** | **DSC** | **NSD** | **HSD** | **NAVE** | **DSC** | **NSD** | **HSD** | **NAVE** |  |
| **L_i_** | 0,44 | 0,33 | 156,53 | 2,04 | 0,41 | 0,36 | 117,99 | 8,56 | 0,50 | 0,40 | 146,48 | 43,69 | 0,60 | 0,57 | 100,20 | 1,54 | 0,08 | 0,07 | 214,80 | 4,91 | 0,36 | 0,37 | 115,25 | 1,84 | 0,40 | 0,35 | 141,88 | 10,43 | 3,17 |
| **E** | 0,45 | 0,36 | 144,22 | 2,04 | 0,39 | 0,35 | 143,63 | 5,00 | 0,40 | 0,35 | 127,44 | 44,74 | 0,54 | 0,48 | 122,92 | 1,51 | 0,29 | 0,19 | 192,66 | 0,59 | 0,33 | 0,25 | 183,49 | 3,18 | 0,40 | 0,33 | 152,39 | 9,51 | 3,67 |
| **FL** | 0,46 | 0,33 | 154,07 | 2,97 | 0,40 | 0,32 | 164,59 | 7,35 | 0,47 | 0,41 | 145,15 | 138,48 | 0,60 | 0,53 | 108,78 | 3,77 | 0,39 | 0,28 | 149,02 | 0,61 | 0,40 | 0,31 | 145,31 | 16,29 | **0,45** | 0,36 | 144,49 | 28,24 | 3,29 |
| **Spec(E)** | 0,46 | 0,36 | 145,24 | 1,79 | 0,41 | 0,36 | 128,10 | 5,64 | 0,43 | 0,37 | 126,60 | 6,73 | 0,56 | 0,51 | 109,91 | 1,37 | 0,30 | 0,19 | 182,49 | 0,63 | 0,34 | 0,28 | 162,96 | 1,29 | 0,42 | 0,35 | 142,55 | **2,91** | 2,58 |
| **Spec(FL)** | 0,46 | 0,34 | 148,15 | 1,37 | 0,42 | 0,36 | 126,06 | 6,18 | 0,51 | 0,45 | 129,80 | 48,29 | 0,61 | 0,58 | 101,55 | 1,86 | 0,28 | 0,22 | 168,17 | 0,68 | 0,37 | 0,31 | 145,14 | 2,24 | 0,44 | **0,38** | **136,48** | 10,10 | **2,29** |

Table A.5: Personalization segmentation evaluation performances among all sites averaged over all sites (all), over manual (man) and automatic (auto) sites.

|  | **avg man** | | | | **avg auto** | | | |
| --- | --- | --- | --- | --- | --- | --- | --- | --- |
|  |  |  |  |  |  |  |  |  |
|  | **DSC** | **NSD** | **HSD** | **NAVE** | **DSC** | **NSD** | **HSD** | **NAVE** |
| **L_i_** | 0,45 | 0,36 | 140,33 | 18,09 | 0,34 | 0,34 | 143,42 | 2,76 |
| **E** | 0,41 | 0,35 | 138,43 | 17,26 | 0,39 | 0,30 | 166,36 | 1,76 |
| **FL** | 0,44 | 0,35 | 154,61 | 49,60 | **0,46** | **0,37** | **134,37** | 6,89 |
| **Spec(E)** | 0,43 | 0,36 | **133,31** | **4,72** | 0,40 | 0,33 | 151,78 | **1,10** |
| **Spec(FL)** | **0,47** | **0,38** | 134,67 | 18,61 | 0,42 | **0,37** | 138,29 | 1,59 |


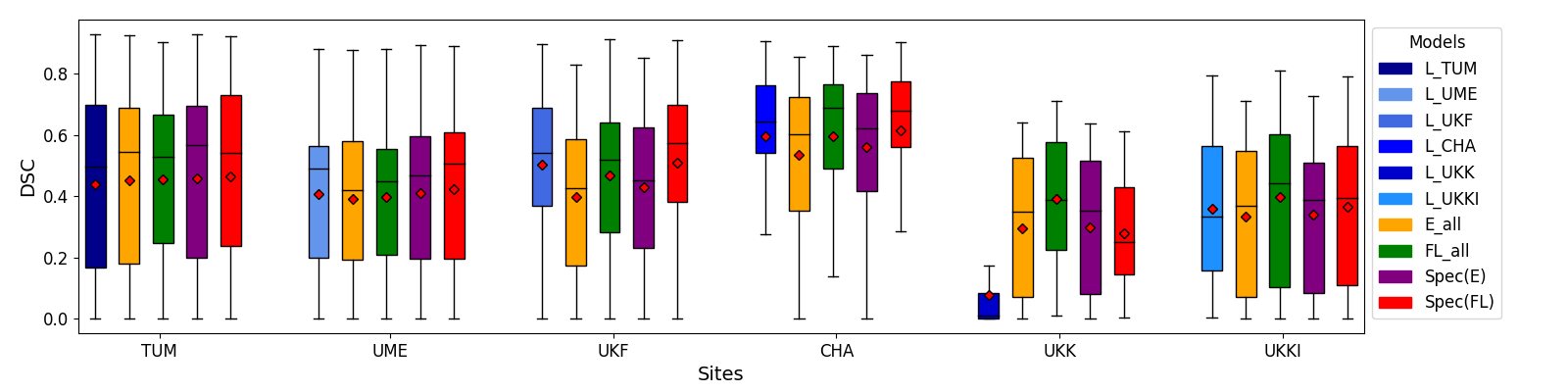


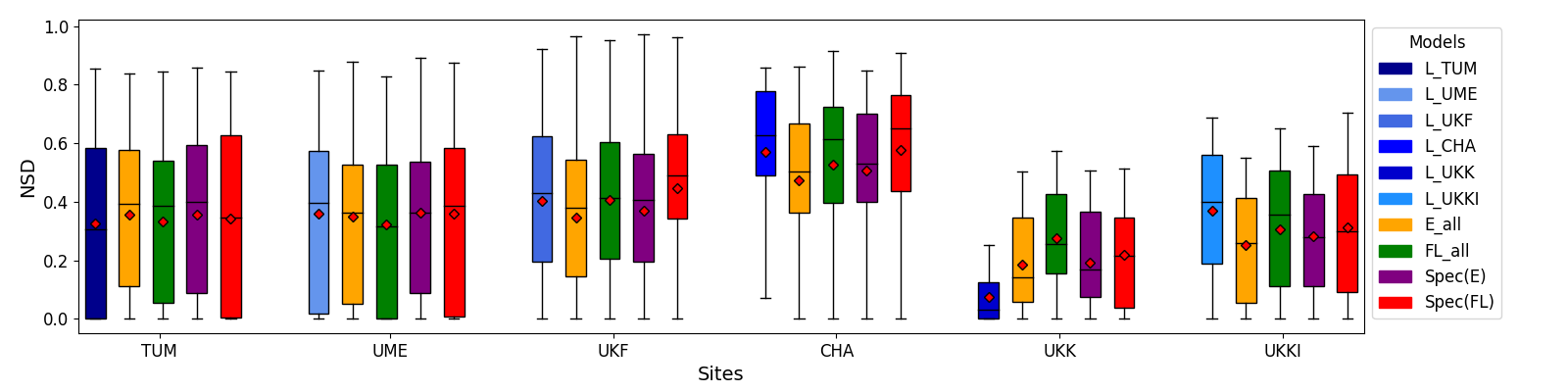


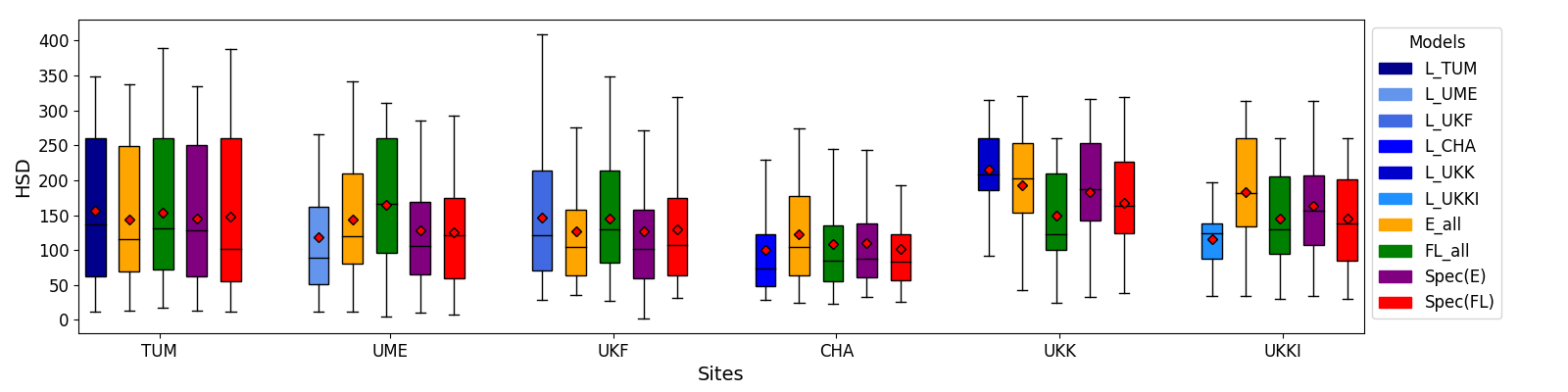


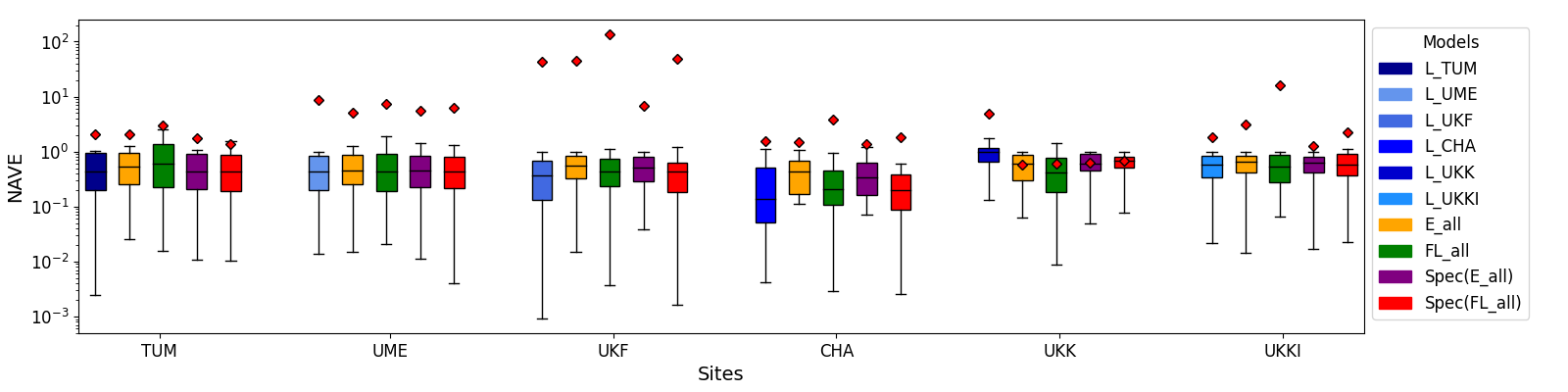


Figure A.6: Boxplots of personalization segmentation evaluation performance among all sites of metrics DSC, NSD, HSD and NAVE.

Table A.6: Generalization without local training segmentation evaluation performance among sites with manually generated annotations (TUM, UME, UKF).

|  | **test data** | | | | | | | | | | | | **avg** | | | | **rank** |
| --- | --- | --- | --- | --- | --- | --- | --- | --- | --- | --- | --- | --- | --- | --- | --- | --- | --- |
|  | **TUM** | | | | **UME** | | | | **UKF** | | | |  |  |  |  |  |
|  | **DSC** | **NSD** | **HSD** | **NAVE** | **DSC** | **NSD** | **HSD** | **NAVE** | **DSC** | **NSD** | **HSD** | **NAVE** | **DSC** | **NSD** | **HSD** | **NAVE** |  |
| **L_TUM_** |  |  |  |  | 0,32 | 0,24 | 149,38 | 11,47 | 0,35 | 0,27 | 149,79 | 73,06 | 0,33 | 0,25 | 149,59 | 42,27 | 3,25 |
| **L_UME_** | 0,38 | 0,29 | 159,35 | 5,47 |  |  |  |  | 0,30 | 0,25 | 169,78 | 875,40 | 0,34 | 0,27 | 164,57 | 440,44 | 4,00 |
| **L_UKF_** | 0,44 | 0,29 | 151,58 | 4,11 | 0,42 | 0,33 | 155,31 | 9,20 |  |  |  |  | **0,43** | 0,31 | 153,45 | **6,65** | 2,75 |
| **E_leave-i-out_** | 0,44 | 0,30 | 142,26 | 3,56 | 0,39 | 0,32 | 137,82 | 8,17 | 0,34 | 0,29 | 143,21 | 12,06 | 0,39 | 0,30 | 141,10 | 7,93 | 2,17 |
| **FL_leave-i-out_** | 0,45 | 0,31 | 138,78 | 3,47 | 0,42 | 0,36 | 134,87 | 6,40 | 0,38 | 0,32 | 149,14 | 18,71 | 0,42 | **0,33** | **140,93** | 9,52 | **1,17** |


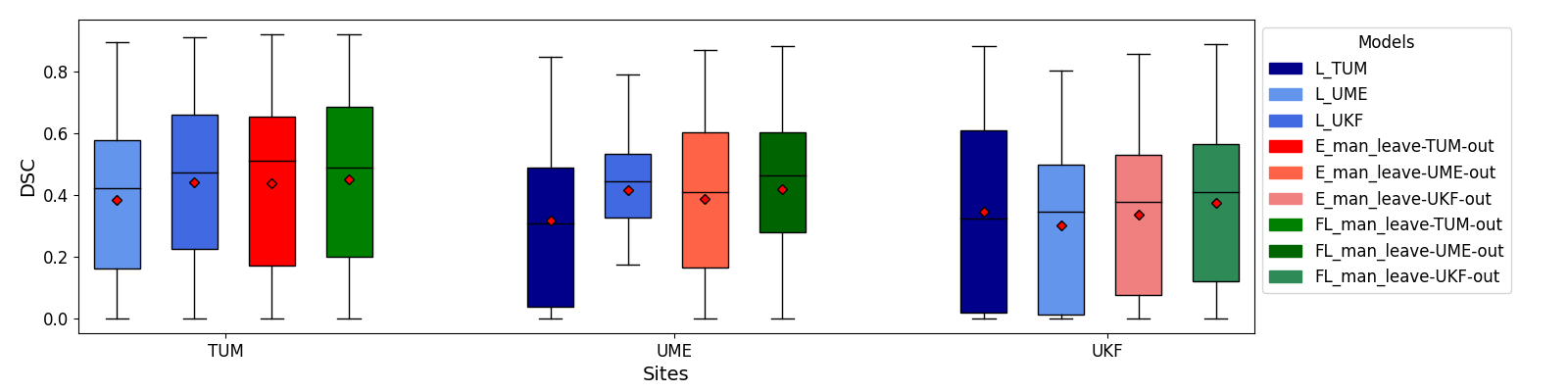


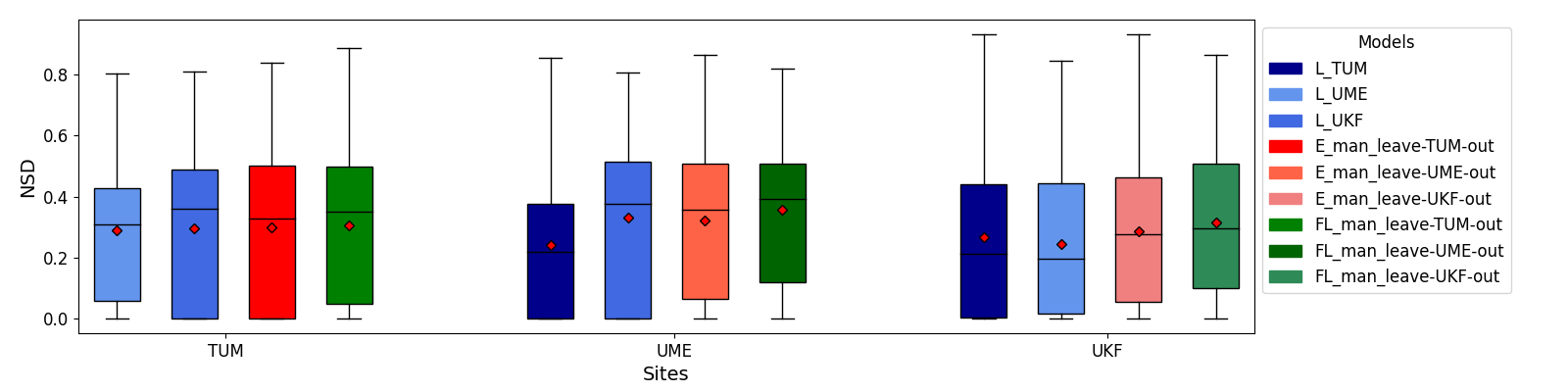


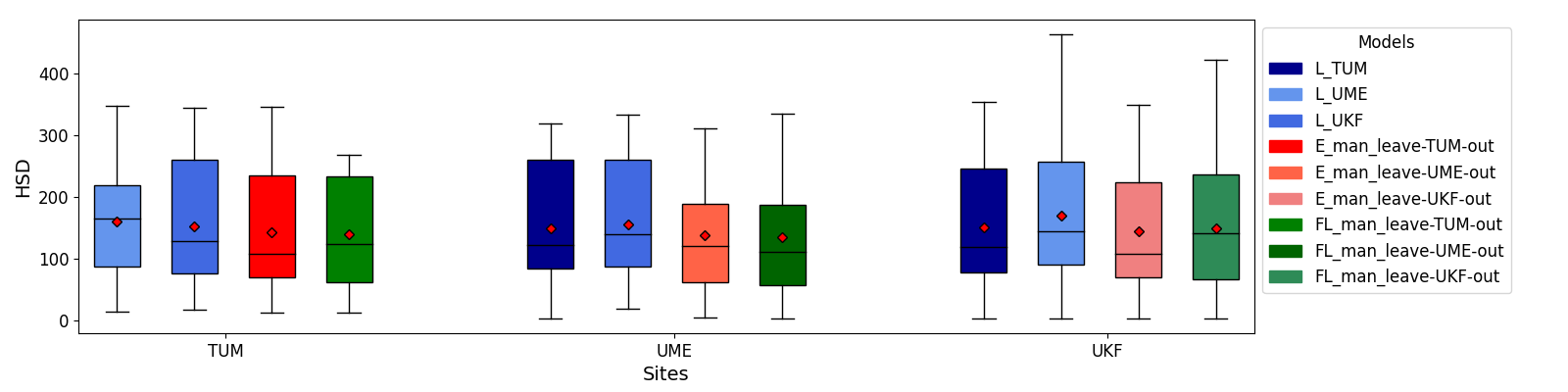


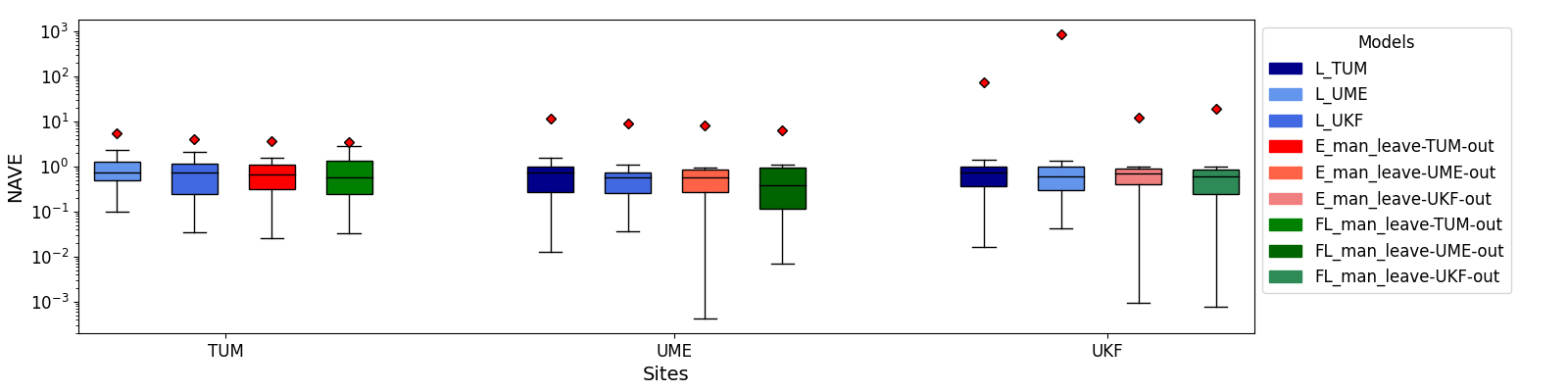


Figure A.7: Boxplots of generalization without local training segmentation evaluation performance among sites with manually generated annotations (TUM, UME, UKF) of metrics DSC, NSD, HSD and NAVE.

Table A.7: Generalization with local training segmentation evaluation performance among sites with manually generated annotations (TUM, UME, UKF).

|  | **test data** | | | | | | | | | | | | **avg** | | | | **rank** |
| --- | --- | --- | --- | --- | --- | --- | --- | --- | --- | --- | --- | --- | --- | --- | --- | --- | --- |
|  | **TUM** | | | | **UME** | | | | **UKF** | | | |  |  |  |  |  |
|  | **DSC** | **NSD** | **HSD** | **NAVE** | **DSC** | **NSD** | **HSD** | **NAVE** | **DSC** | **NSD** | **HSD** | **NAVE** | **DSC** | **NSD** | **HSD** | **NAVE** |  |
| **L_TUM_** | 0,44 | 0,33 | 156,53 | 2,04 | 0,32 | 0,24 | 149,38 | 11,47 | 0,35 | 0,27 | 149,79 | 73,06 | 0,37 | 0,28 | 151,90 | 28,86 | 5,73 |
| **L_UME_** | 0,38 | 0,29 | 159,35 | 5,47 | 0,41 | 0,36 | 117,99 | 8,56 | 0,30 | 0,25 | 169,78 | 875,40 | 0,36 | 0,30 | 149,04 | 296,48 | 5,53 |
| **L_UKF_** | 0,44 | 0,29 | 151,58 | 4,11 | 0,42 | 0,33 | 155,31 | 9,20 | 0,50 | 0,40 | 146,48 | 43,69 | 0,45 | 0,34 | 151,12 | 19,00 | 4,93 |
| **E** | 0,45 | 0,36 | 144,22 | 2,04 | 0,39 | 0,35 | 143,63 | 5,00 | 0,40 | 0,35 | 127,44 | 44,74 | 0,41 | 0,35 | 138,43 | 17,26 | 3,33 |
| **FL_leave-i-out_** | 0,45 | 0,31 | 138,78 | 3,47 | 0,42 | 0,36 | 134,87 | 6,40 | 0,38 | 0,32 | 149,14 | 18,71 | 0,42 | 0,33 | 140,93 | 9,52 | 3,87 |
| **Spec(E)** | 0,47 | 0,34 | 144,50 | 2,60 | 0,42 | 0,38 | 104,75 | 8,43 | 0,46 | 0,39 | 123,96 | 3,06 | 0,45 | **0,37** | 124,41 | 4,70 | 2,33 |
| **Spec(FL_leave-i-out_)** | 0,48 | 0,33 | 136,57 | 0,98 | 0,44 | 0,39 | 112,69 | 6,79 | 0,45 | 0,39 | 127,19 | 3,19 | **0,46** | **0,37** | **125,48** | **3,65** | **2,27** |


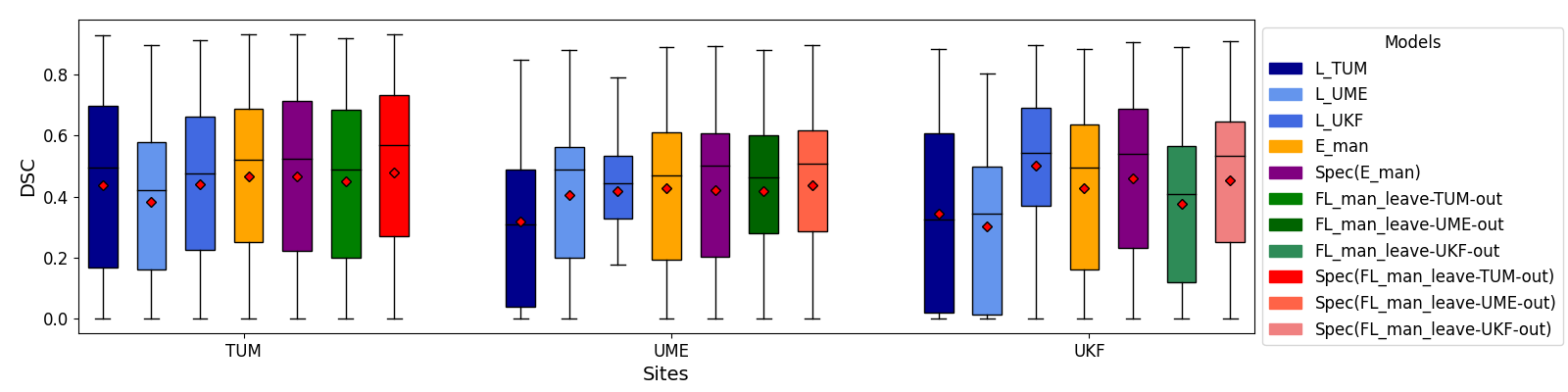


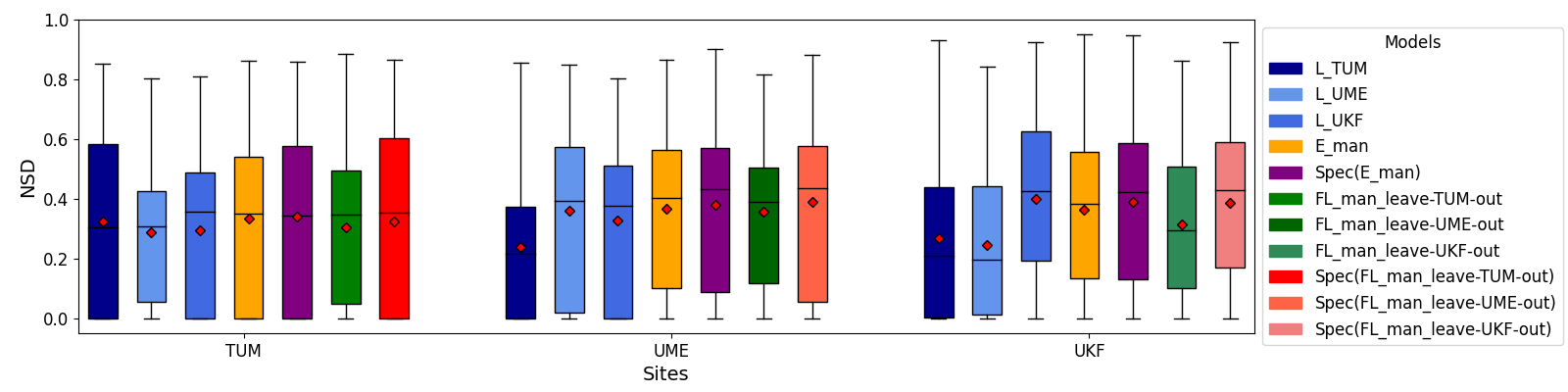


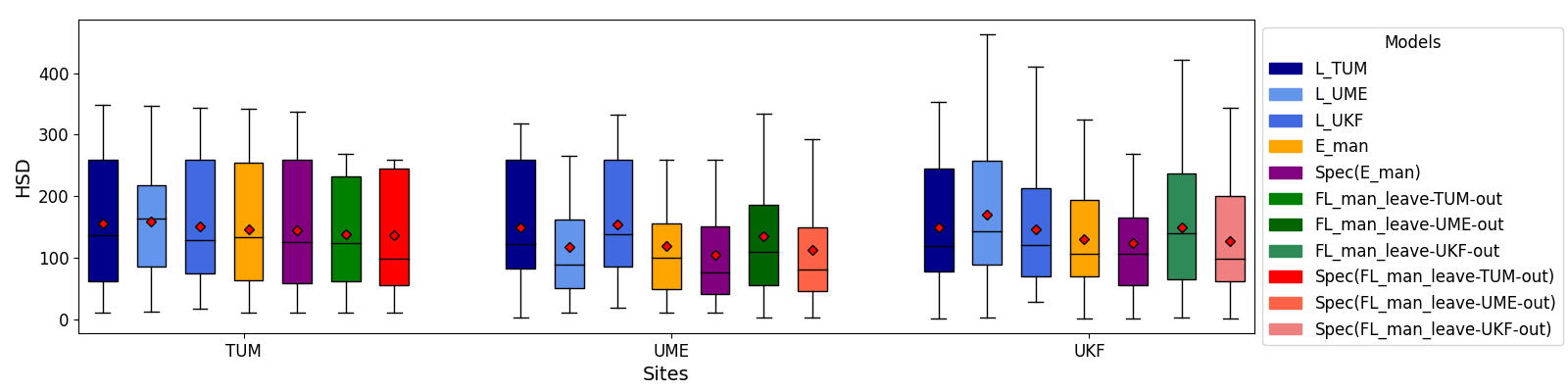


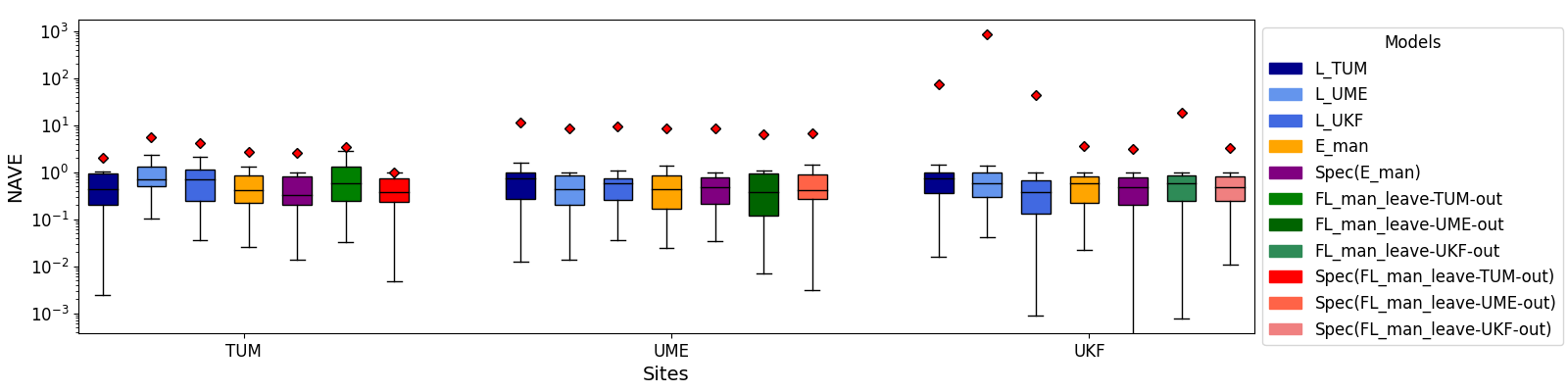
Figure A.8: Boxplots of generalization without local training segmentation evaluation performance among sites with manually generated annotations (TUM, UME, UKF) of metrics DSC, NSD, HSD and NAVE.
